# Assessing the structural boundaries of broadly reactive antibody interactions with diverse H3 influenza hemagglutinin proteins

**DOI:** 10.1128/jvi.00453-25

**Published:** 2025-08-14

**Authors:** John V. Dzimianski, Kaito A. Nagashima, Joseph M. Cruz, Giuseppe A. Sautto, Sara M. O’Rourke, Vitor H. B. Serrão, Ted M. Ross, Jarrod J. Mousa, Rebecca M. DuBois

**Affiliations:** 1 Department of Biomolecular Engineering, University of California Santa Cruz 8787, Santa Cruz, California, USA; 2 Center for Vaccines and Immunology, University of Georgia 822673, Athens, Georgia, USA; 3 Department of Infectious Diseases, University of Georgia 551782, Athens, Georgia, USA; 4 Florida Research and Innovation Center, Cleveland Clinic 587918 , Port St. Lucie, Florida, USA; 5 Biomolecular Cryo-Electron Microscopy Facility, University of California Santa Cruz 8787, Santa Cruz, California, USA; 6 Department of Chemistry and Biochemistry, University of California Santa Cruz 8787, Santa Cruz, California, USA; 7 Department of Biomedical Sciences, Florida State University College of Medicine 465938, Tallahassee, Florida, USA; Emory University School of Medicine, Atlanta, Georgia, USA

**Keywords:** influenza, H3N2, hemagglutinin, COBRA, hemagglutinin vaccine, universal influenza vaccine, broadly neutralizing antibody, antigenic drift, structural biology, cryo-EM, X-ray crystallography, biolayer interferometry

## Abstract

Influenza virus infections are an ongoing seasonal disease burden and a persistent pandemic threat. Formulating successful vaccines remains a challenge due to accumulating mutations in circulating strains, necessitating the development of innovative strategies to combat present and future viruses. One promising strategy for attaining greater vaccine effectiveness and longer-lasting protection is the use of computationally optimized broadly reactive antigens (COBRAs). The COBRA approach involves *in silico* antigen design by generating iterative, layered consensus sequences based on current and historic viruses. Antigens designed by this process show a greater breadth of antibody-mediated protection compared to wild-type antigens, with effectiveness that often extends beyond the sequence design space of the COBRA. In particular, the use of COBRA hemagglutinin (HA) proteins has led to the discovery of broadly reactive antibodies that are suggestive of their therapeutic potential. Understanding the extent to which these antibodies are effective is key to assessing the resilience of vaccine-induced immunity to diverging influenza strains. To investigate this, we tested the binding of broadly reactive antibodies with a diverse panel of H3 HA proteins. Using cryo-electron microscopy, we defined the molecular characteristics of binding for these antibodies at the paratope-epitope interface. Through sequence and structural comparisons, we observed the correlative patterns between antibody affinity and antigen structure. These data shed light on the breadth and limitations of broadly reactive antibody responses in the context of an ever-changing landscape of influenza virus strains, yielding insights into strategies for universal vaccine design.

**IMPORTANCE:**

Formulating effective influenza vaccines remains a challenge due to a constantly changing landscape of circulating viruses. This is particularly true for H3N2 viruses that undergo a high degree of antigenic drift. Several new vaccine designs can elicit broadly neutralizing antibodies that are effective against a range of influenza strains. More insight is needed, however, into how resilient these antibodies will be to future strains that evolve in the context of this selective pressure. Here, we measured the precise binding characteristics of three broadly neutralizing antibodies to 18 different hemagglutinin (HA) proteins representing almost 50 years of virus evolution. Using single-particle cryo-electron microscopy and X-ray crystallography, we determined the structural characteristics of the epitopes bound by these antibodies and identified specific amino acids that greatly impact the effectiveness of these antibodies. This provides important insights into the longevity of antibody efficacy that can help guide design choices in next-generation vaccines.

## INTRODUCTION

Influenza presents a persistent public health threat. Since the identification of a virus as the causative agent of the 1918 influenza pandemic, various strains of influenza A virus (IAV) and influenza B virus (IBV) have caused regular outbreaks and epidemics, with some leading to IAV pandemics in 1957, 1968, and 2009 (1). The introduction of successful influenza vaccines in the 1940s led to a new era in public health in which influenza-caused diseases could be mitigated (2). However, due to the emergence of new strains from zoonotic spillover, virus reassortment or recombination (“genetic shift”), and accumulation of point mutations (“genetic drift”), influenza vaccines are susceptible to strain mismatches that lower their effectiveness (1). Despite active epidemiological monitoring to assess strain prevalence and the use of multivalent vaccines to cover divergent circulating viruses, annual vaccine efficacy generally ranges from 30% to 60% (3). As a result, there is a need for the development of new vaccine approaches that yield a greater breadth and duration of protection (4).

**Fig 1:**
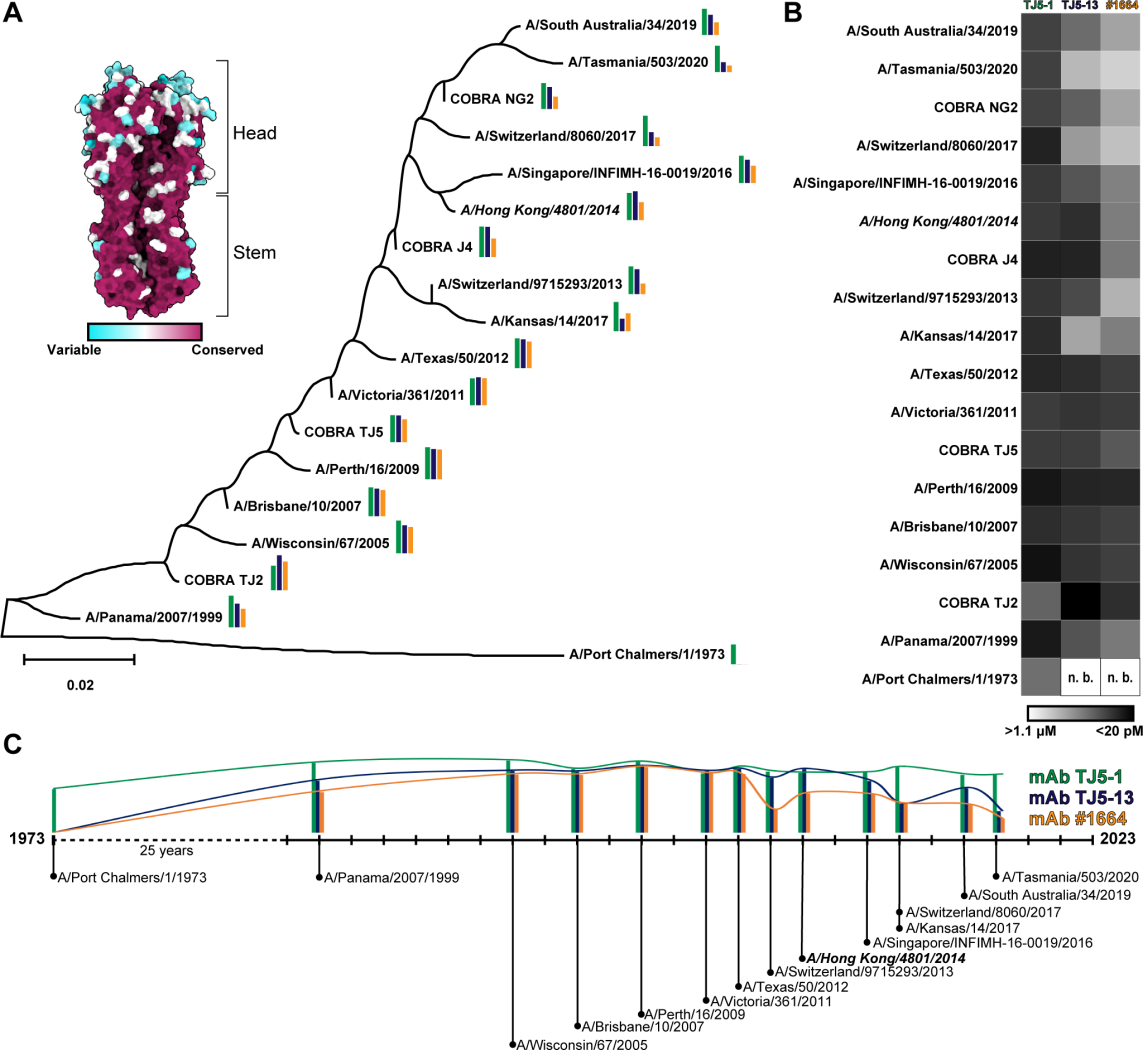
Impact of genetic drift on antibody binding over time. (**A**) Sequence-based divergence of H3 proteins over time. A model of COBRA HA TJ2 was surface colored based on residue-by-residue conservation in ChimeraX (7). The tree was generated in MEGA 11, with evolutionary history inferred using the Maximum Likelihood method and JTT matrix-based model (8–10). Relative binding for the HA proteins with each antibody is shown as colored bars, with green representing TJ5-1, blue representing TJ5-13, and orange representing #1664. The relevant vaccine strain, A/Hong Kong/4801/2014, is denoted by italicized font. (**B**) Heatmap representing the relative binding affinities, as determined by biolayer interferometry, of the TJ5-1, TJ5-13, and #1664 antibodies with each HA tested. The relative scale ranges from white (no measurable binding) to black (maximum measured binding). (**C**) Relative binding levels for each antibody for different strains, plotted over time. For simplicity, affinity values for virus strains from the same year were averaged together.

The current primary metric used to infer immune protection against influenza, in the absence of virus challenge, is the presence of neutralizing antibodies. Neutralizing antibodies target the hemagglutinin (HA), a trimeric protein containing “stem” (also known as the “stalk”) and “head” domains in each protomer. The head domain includes the Receptor Binding Site (RBS), making this domain the primary target of neutralizing antibodies by blocking virus attachment to host cells. The stem domain contains the fusion peptide, which is targeted by a smaller subset of neutralizing antibodies that prevent membrane fusion. The degree to which these regions are conserved is inversely proportional to the number of antibodies that target them. The immunodominant head domain, while having specific regions such as the RBS that are highly conserved due to functional constraints, is generally more variable than the immunosubdominant stem domain (Fig. 1A). Antibodies that target the more highly conserved regions of HA have been a particular focus of interest in the context of antivirals and vaccination, and several broadly reactive antibodies—with and without neutralizing activity—have been the focus of discovery efforts and molecular characterization (5, 6).

Given the potential usefulness of broadly reactive antibodies in providing broad and durable protection against diverse influenza strains, several next-generation vaccine designs seek to take advantage of conserved elements in the HA structure to elicit these types of antibodies (11). Some employ a stem-focused approach that excludes or diminishes the immunogenicity of the head domain, such as stem-presenting nanoparticles (12) and chimeric HA proteins that combine the stem domain of interest with heterosubtypic head domains (13). Other designs, such as centralized conserved (14) and computationally optimized broadly reactive antigen (COBRA) HA proteins (15), retain the subtype-specific structural architecture in both domains while seeking to accentuate the conserved features common to circulating strains. A distinctive feature of the COBRA approach is the use of existing sequence information in a layered, iterative manner to both incorporate strongly conserved features while mitigating bias due to overrepresented strains in sequence databases (15). This permits the generation of multiple candidate antigens via different computational permutations that can be screened for optimal reactivity. From this computational approach, COBRA HA proteins have been designed from IAV H1, H2, H3, H5, H7, and IBV HA sequences that provide vaccine-based protection in animal models against a more diverse range of viruses compared to homosubtypic wild-type HA antigens (15–20). While this can partially be accounted for by diverse polyclonal antibody responses, COBRA HA proteins also mediate protection by eliciting broadly neutralizing antibodies that target highly conserved epitopes (21, 22).

While much work has gone into understanding the features of broadly neutralizing antibodies that drive their breadth of activity, less has been done to explicitly examine the limitations of their interaction with viral antigens. In general, the limitations have mostly been viewed from the standpoint of subtype specificity or genetic shift within a subtype. The susceptibilities of these antibodies to changes in future strains by genetic drift are less well understood. This is particularly relevant for H3N2 viral strains, which undergo a faster rate of antigenic change compared to other influenza viruses circulating within human populations (23, 24). As the elicitation of broadly reactive antibodies is a general goal of next-generation vaccines, a better understanding of their resilience or vulnerabilities to genetic drift is needed, particularly given the potential outlook of increased selective pressure on conserved regions of the HA.

To explore how genetic drift in H3N2 viruses impacts broadly neutralizing antibodies, we assessed the precise binding characteristics of three broadly neutralizing monoclonal antibodies—one stem-binding (TJ5-1) and two head-binding (TJ5-13 and #1664)—with divergent H3 HA proteins. Using biolayer interferometry, the affinity and binding kinetics were assessed for each antibody with 18 recombinant H3 HA proteins representing almost 50 years of sequence space. This revealed that the stem-binding TJ5-1 antibody possesses a greater resilience to current patterns of genetic drift compared to TJ5-13 and #1664. The structural features of binding for each antibody with the COBRA HA NG2 protein were elucidated by single-particle cryo-electron microscopy (cryo-EM), revealing the critical interfaces of these antibodies with HA. Through structural comparisons with other HA proteins, including novel crystal structures of COBRA HAs TJ2 and J4, we identified the residues primarily responsible for the variable binding patterns observed by biolayer interferometry.

## RESULTS

### Broadly neutralizing antibodies show variable sensitivities to genetic drift

Previous studies identified the broadly neutralizing monoclonal antibodies (mAbs) #1664, TJ5-1, and TJ5-13 from human cohorts that received a commercial split inactivated vaccine, containing A/Hong Kong/4801/2014 as the H3N2 component, during the 2016–2017 (mAb #1664) or 2017–2018 (mAbs TJ5-1 and TJ5-13) northern hemisphere influenza seasons (Tables 1 and 2) (25, 26). These investigations revealed that although these mAbs showed general patterns of potent reactivity against various seasonal H3N2 strains, their effectiveness was sometimes diminished for a subset of viruses. To gain a more precise understanding of the temporal relationships between H3N2 strains and antibody binding, we used biolayer interferometry (BLI) to measure the affinity and binding kinetics of these antibodies with a panel of diverse recombinant HA proteins (Fig. 1; Fig. S1 and S2; Table 3). This included representation by 14 virus strains ranging from 1973 to 2020, as well as four COBRA HA proteins designed from different sequence eras within the years 2002–2019 (27, 28).

**TABLE 1:** Germline lineage and CDR sequences for antibody heavy chains^*a*^

mAb	HC VDJ subfamilies	HCDR1 sequence	HCDR2 sequence	HCDR3 sequence	Reference
TJ5-1	*V_H_1-2* *D_H_5-12* *J_H_6*	GYTFTGFY	INPHSGDT	VKNDIVLGMGV	(25)
TJ5-13	*V_H_1-69* *D_H_3-22* *J_H_4*	GDTFTTYSG	VLPNFGSP	TETGAYNSVGYFPYFQFR	(25)
#1664	*V_H_1-69-2* D_H_5-24*01 J_H_6*02	GYTFSDYF	VDVDNGEV	ASTTPRGGNPSVYNYFFVDV	(26)

^*a*^ Underlined = interface residues.

**TABLE 2:** Germline lineage and CDR sequences for antibody light chains^*a*^

mAb	LC VJ subfamilies	LCDR1 sequence	LCDR2 sequence	LCDR3 sequence	Reference
TJ5-1	*V_L_1-40* *J_L_1*	SSNIGAGYN	GDN	QSYDSSLNAYV	(25)
TJ5-13	*V_L_1-40* *J_L_1*	SSNIGADYD	GNN	QSFDKSLSGSFV	(25)
#1664	V_L_4-60*03 J_L_2*01	SGHDNYI	VGAGGTY	CETWDSKTVF	(26)

^*a*^ Underlined = interface residues.

**TABLE 3:** Kinetics of antibody binding with HA

	mAb TJ5-1			mAb TJ5-13			mAb #1664		
Hemagglutinin	Average K_D_ (nM)	Average ka (M^−1^s^−1^)	Average kd (s^−1^)	Average K_D_ (nM)	Average ka (M^−1^s^−1^)	Average kd (s^−1^)	Average K_D_ (nM)	Average ka (M^−1^s^−1^)	Average kd (s^−1^)
SA/2019	1.09	2.96 × 10^5^	3.21 × 10^−4^	8.69	5.81 × 10^3^	5.05 × 10^−5^	119	4.60 × 10^3^	4.76 × 10^−4^
Tas/2020	1.05	1.41 × 10^5^	1.50 × 10^−4^	330	1.87 × 10^3^	5.81 × 10^−4^	1050	2.78 × 10^3^	2.95 × 10^−3^
COBRA NG2	1.19	2.58 × 10^5^	2.48 × 10^−4^	4.58	7.45 × 10^3^	3.17 × 10^−5^	136	4.53 × 10^3^	6.09 × 10^−4^
Switz/2017	0.25	3.89 × 10^5^	9.60 × 10^−5^	79.2	3.71 × 10^3^	2.66 × 10^−4^	483	3.50 × 10^3^	1.69 × 10^−3^
Sing/2016	0.71	1.89 × 10^5^	1.36 × 10^−4^	2.90	1.33 × 10^5^	3.85 × 10^−4^	23.4	9.84 × 10^4^	2.30 × 10^−3^
HK/2014	0.77	1.95 × 10^5^	1.48 × 10^−4^	0.44	2.02 × 10^5^	8.85 × 10^−5^	19.6	7.88 × 10^4^	1.55 × 10^−3^
COBRA J4	0.23	2.84 × 10^5^	6.67 × 10^−5^	0.26	1.20 × 10^5^	3.06 × 10^−5^	15.2	3.01 × 10^4^	4.54 × 10^−4^
Switz/2013	0.63	3.03 × 10^5^	1.90 × 10^−4^	1.71	9.86 × 10^4^	1.66 × 10^−4^	254	1.50 × 10^4^	3.79 × 10^−3^
Kan/2017	0.39	2.33 × 10^5^	9.06 × 10^−5^	133	2.48 × 10^4^	3.28 × 10^−3^	20.3	9.45 × 10^5^	1.89 × 10^−2^
Tex/2012	0.30	3.07 × 10^5^	8.93 × 10^−5^	0.42	2.57 × 10^5^	9.53 × 10^−5^	0.93	9.83 × 10^4^	9.08 × 10^−5^
Vic/2011	0.86	2.28 × 10^5^	1.95 × 10^−4^	0.59	2.18 × 10^5^	1.28 × 10^−4^	0.84	1.19 × 10^5^	8.98 × 10^−5^
COBRA TJ5	0.82	2.27 × 10^5^	1.57 × 10^−4^	0.90	1.39 × 10^5^	1.26 × 10^−4^	3.42	5.92 × 10^4^	2.01 × 10^−4^
Perth/2009	0.14	2.66 × 10^5^	3.66 × 10^−5^	0.27	2.26 × 10^5^	6.03 × 10^−5^	0.31	1.08 × 10^5^	3.36 × 10^−5^
Bris/2007	0.43	2.17 × 10^5^	9.26 × 10^−5^	0.66	2.12 × 10^5^	1.38 × 10^−4^	1.06	9.41 × 10^4^	8.36 × 10^−5^
Wis/2005	0.11	2.00 × 10^5^	4.54 × 10^−5^	0.58	2.08 × 10^5^	1.20 × 10^−4^	1.03	1.59 × 10^5^	1.50 × 10^−4^
COBRA TJ2	5.64	2.30 × 10^5^	3.48 × 10^−3^	0.05	1.59 × 10^5^	8.68 × 10^−6^	0.45	1.21 × 10^5^	5.44 × 10^−5^
Pan/1999	0.16	3.15 × 10^5^	4.97 × 10^−5^	1.14	1.14 × 10^5^	3.04 × 10^−4^	16.4	1.09 × 10^5^	1.79 × 10^−3^
PC/1973	10.1	4.81 × 10^5^	4.83 × 10^−4^	NB^*a*^	NB	NB	NB	NB	NB

^*a*^ NB, no binding.

Assessment of the overall trends reveals that the HA stem-binding mAb TJ5-1 possesses a consistently strong binding profile over time (Fig. 1B and C). From A/Panama/2007/1999 to A/South Australia/34/2019, the affinities of TJ5-1 for different HA proteins remain in the low nanomolar to picomolar range. Even the worst binder, A/Port Chalmers/1/1973, still shows an affinity of ~10 nM with only a moderate reduction in the individual association and dissociation rates (Table 3). This points to an epitope that has shown little to no change since 1999. By contrast, the HA head-binding mAbs TJ5-13 and #1664 show a wide range of binding profiles, with no measurable binding for A/Port Chalmers/1/1973 and picomolar binding for HA proteins from 2002 to 2012, such as COBRA HA TJ2 and A/Perth/16/2009. Both show a general trend of lower reactivity to HAs from strains after 2013, with mAb #1664 impacted to a greater extent. Interestingly, while there are general similarities in the trends of binding, there are specific cases in which they differ, most notably in the case of A/Switzerland/9715293/2013. While TJ5-13 retains moderate binding for this HA, the affinity of #1664 is much weaker. Beyond this, the main differences in the two antibodies are a generally tighter affinity of TJ5-13 that can be partly attributed to slower rates of dissociation compared to #1664. Overall, the BLI data suggest that mAb TJ5-1 binds a highly conserved epitope that shows little change during genetic drift, whereas TJ5-13 and #1664 may bind epitopes that are affected in similar ways by recent patterns of virus divergence.

### TJ5-1 binds a highly conserved epitope and inhibits activation of the fusion peptide

**Fig 2:**
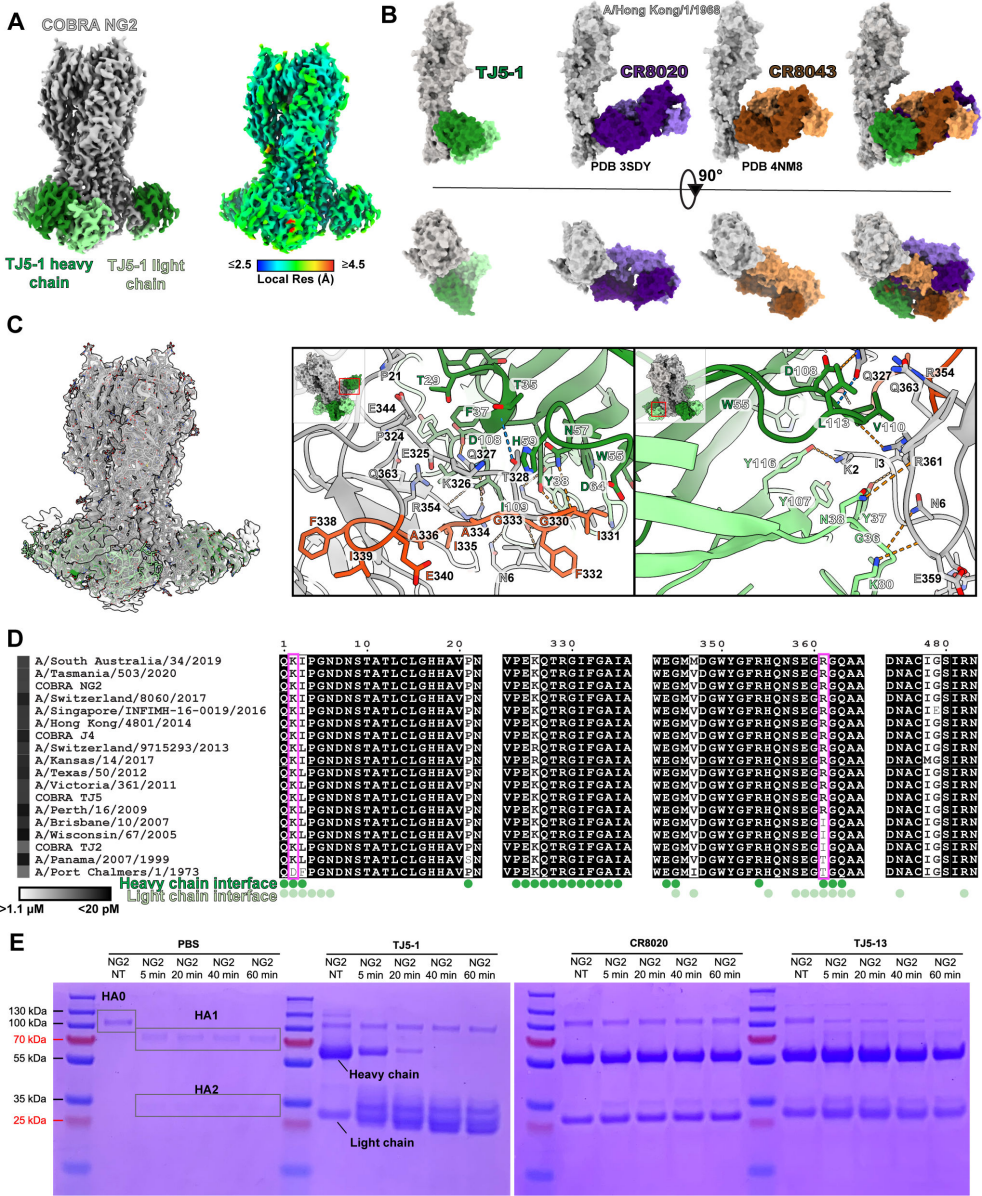
Single-particle cryo-EM structure of COBRA HA NG2 bound by the TJ5-1 Fab. (**A**) Cryo-EM reconstructed map of the antigen-Fab complex, color-coded based on protein chains or local resolution estimation. (**B**) Comparison of stem-targeting antibodies bound to H3 proteins. (**C**) Analysis of the binding interface in the model built into the map. The overall fit of the model in the map is shown in the left panel, with close-up views of the interface shown in the middle and right panels. Overall viewing orientations and regions of focus within the model are indicated by inset images. The heavy chain of the antibody is shown in dark green, the light chain in light green, COBRA HA NG2 in light gray, and the fusion peptide of COBRA HA NG2 in red-orange. Hydrogen bonds are shown by dashed lines, with those meeting the strict criteria in ChimeraX colored blue and those identified under more permissive criteria colored orange. (**D**) Sequence alignment of the HA proteins used in this study, with residue positions colored based on sequence conservation with ESPript (31). Perfectly conserved residues are shown in white lettering on a black background, those that are partly conserved as bold black text on a white background, and those that are not conserved as normal black text on a white background. The relative level of TJ5-1 binding with each HA is shown to the left of each virus label as a heat map, ranging from light gray (moderate binding) to black (maximal binding). HA residues that are within the buried surface area of the interface with TJ5-1, based on PDBePISA (32), are indicated, with residue positions showing the greatest variation boxed in magenta. (**E**) Trypsin activation assay of COBRA HA NG2, visualized by SDS-PAGE with Coomassie staining. The bands corresponding to the HA0 and HA1/HA2 states of COBRA HA NG2 are indicated, as well as the bands corresponding to the heavy and light chains of IgG. The TJ5-1 heavy chain shows partial degradation in the presence of trypsin, with evidence of Fc removal but probable retention of the Fab domain. NT, no trypsin control.

To better understand the resilience of mAb TJ5-1 to genetic drift, we characterized the structural features of binding by single particle cryo-EM analysis of the fragment antigen binding (Fab) region of TJ5-1 in complex with the COBRA HA NG2 (Fig. 2; Fig. S3; Table 4). The resulting map at 3.24 Å resolution revealed that TJ5-1 binds NG2 at a membrane-proximal epitope in the stem domain (Fig. 2A). While possessing a footprint that overlaps the well-characterized CR8020 and CR8043 stem-binding antibodies (29, 30), TJ5-1 binds in a different orientation. Compared to these other two antibodies, TJ5-1 is rotated with the light and heavy chains positioned in a planar rather than vertical pose relative to the HA (Fig. 2B). This results in a binding interface that involves the light chain to a greater degree, overall encompassing a greater number of residues in the buried surface area.

**TABLE 4:** Cryo-EM data collection, refinement, and validation statistics

	NG2:TJ5-1 (EMD-47024) (PDB 9DN2)	NG2:TJ5-13 (EMD-44305) (PDB 9B7G)	NG2:#1664 (EMD-47071) (PDB 9DO2)
Data collection and processing			
Magnification	22,500	105,000	190,000
Voltage (kV)	300	300	200
Electron exposure (e–/Å^2^)	60.216	45.8	57.00
Defocus range (μm)	0.8-2.4	0.5-2.1	0.9-2.0
Pixel size (Å)	1.093	0.835	0.526
Symmetry imposed	C3	C3	C1
Initial particle images (no.)	1,039,511 (selected 2D classes)	298,059 (selected 2D classes)	110,663 (selected 2D classes)
Final particle images (no.)	248,400 (final 3D refinement)	70,400 (final 3D refinement)	110,663 (final 3D refinement)
Map resolution (Å)	3.24	2.61	3.45
FSC threshold	0.143	0.143	0.143
Refinement			
Initial model used	PDB 6WZT (HA), PDB 4Q9Q (Fab)	SwissModel (HA), SabPred (Fab)	PDB 6WZT (HA), PDB 4Q9Q (Fab)
Model resolution (Å)	3.6	2.9	3.8
FSC threshold	0.5	0.5	0.5
Map sharpening *B* factor (Å^2^)	−112.6	−63.3	−90.4
Model composition			
Non-hydrogen atoms	17,406	13,800	9,330
Protein residues	2,148	1,704	1,109
Ligands	48	42	45
*B* factors (Å^2^)			
Protein	75.26	41.27	84.37
Ligand	87.81	41.51	117.19
R.m.s. deviations			
Bond lengths (Å)	0.002	0.002	0.002
Bond angles (°)	0.594	0.466	0.588
Validation			
MolProbity score	1.74	1.30	1.75
Clashscore	4.07	3.36	5.10
Poor rotamers (%)	1.96	0.81	2.05
CaBLAM outliers (%)	2.84	2.54	2.91
Q-score (overall)	0.466	0.571	0.462
Ramachandran plot			
Favored (%)	95.35	96.96	96.41
Allowed (%)	4.65	3.04	3.59
Disallowed (%)	0.00	0.00	0.00

A deeper analysis of the model built into the map reveals more details on TJ5-1’s distinct characteristics of binding (Fig. 2C and D). The heavy chain of TJ5-1 presents a predominantly hydrophobic surface at the points of contact in the complementary determining region (CDR) loops—F37 and Y38 in CDR loop 1, W55 in loop 2, and a series of isoleucine, valine, and leucine residues in positions 109–113 of loop 3. Interestingly, while there are a few hydrophobic residues present in the HA interface, such as I331 within the fusion peptide, most of the amino acids within the buried surface area contain charged or polar residues such as threonine, lysine, arginine, asparagine, glutamine, and glutamate. Some of these residues accommodate the antibody by adopting side chain orientations that position the polar head groups away from the interface, permitting the locally hydrophobic methylene groups to pack against the hydrophobic surface of the antibody. In addition, however, the heavy chain also forms polar contacts in the interface, including hydrogen bonding between D108 (heavy chain) and Q327 (HA), the backbone of I109 and Q327, the backbone of I109 with R361, and the side chains of N57 and Y38 with G330 (Fig. 2C). Similarly, the light chain of TJ5-1 also contributes a mixture of hydrophobic and polar interactions. The main contribution appears to be a pocket in the light chain formed by Y37, Y107, and Y116 that accommodates the aliphatic side chain of HA K2 through a stabilizing hydrogen bond with Y116. Interestingly, this position contains an aspartate in the HA protein from A/Port Chalmers/1/1973 (Fig. 2D). This likely accounts for the substantially lower affinity in binding observed for this HA compared to the others, as the less flexible aspartate would be unable to reposition its charged group to the same degree. Finally, there are polar contacts in the form of a hydroxyl-backbone hydrogen bond interaction between Y37 (light chain) with I3 (HA), K80 with the backbone of E359, and the backbone of G36 with N6.

Given the binding position of TJ5-1 and its direct contact with the fusion peptide, we hypothesized that this antibody would be able to interfere with the conversion of the HA from the HA0 precursor to the HA1/HA2 primed state. To test this, we assessed the ability of this antibody to inhibit activation of the HA by trypsin (Fig. 2E). Similar to the established CR8020 antibody, TJ5-1 strongly diminishes the conversion of COBRA HA NG2 from the HA0 to HA1/HA2 state. By contrast, the head-binding mAb TJ5-13 antibody shows a conversion from the HA0 to the HA1/HA2 state over time. This suggests that TJ5-1 achieves virus neutralization by preventing maturation of the HA and/or restricting the mobility of the fusion peptide to prevent membrane fusion.

### TJ5-13 and #1664 bind similar epitopes involving the RBS

**Fig 3:**
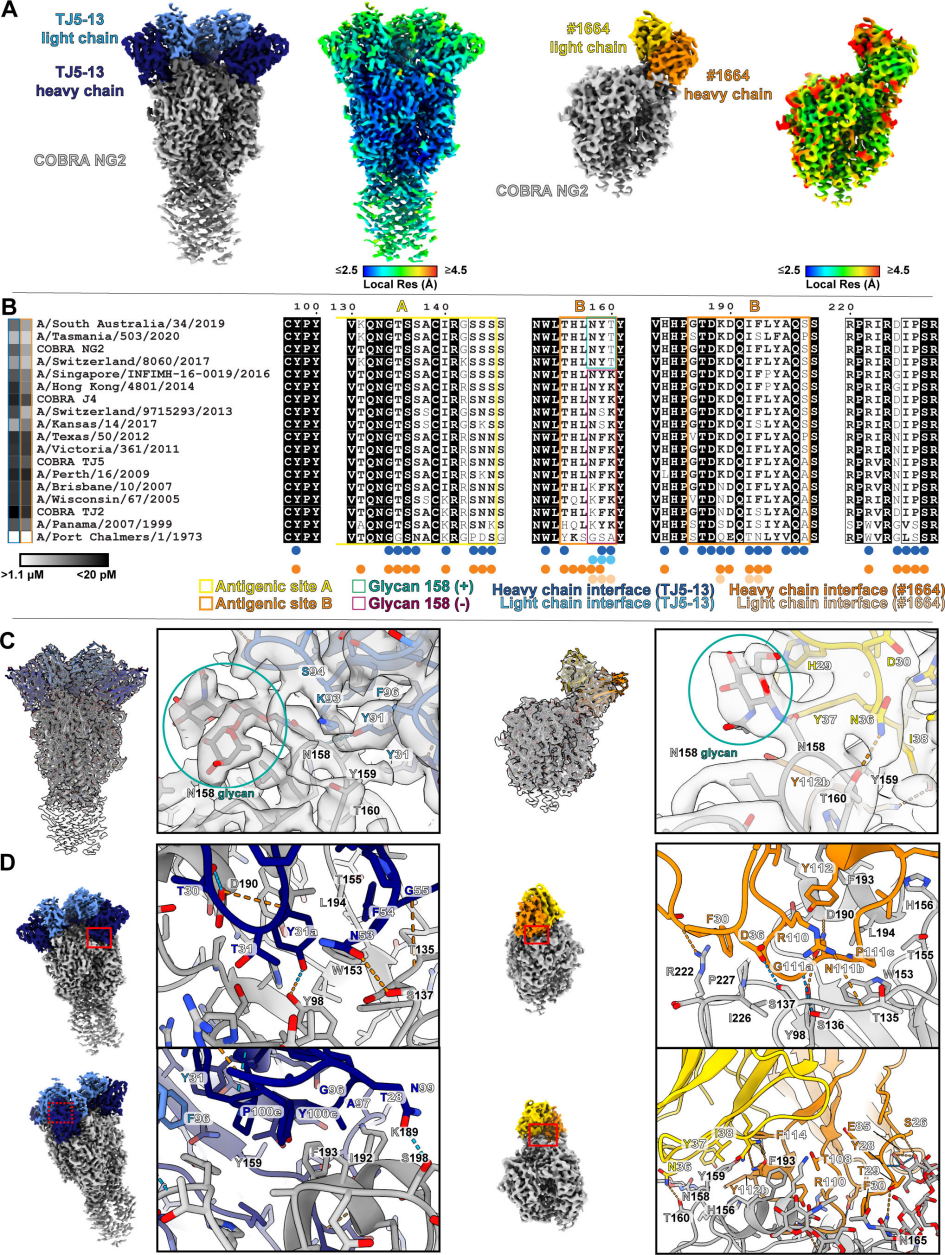
Single-particle cryo-EM structures of COBRA HA NG2 bound to the TJ5-13 and #1664 Fabs. (**A**) Overall cryo-EM reconstructed maps, color-coded based on protein chains or local resolution. (**B**) Sequence alignment of the HA proteins used in this study, with residue positions colored based on sequence conservation with ESPript (31). Residues that are perfectly conserved are shown in white lettering on a black background, those that are partly conserved as bold black text on a white background, and those that are not conserved as normal black text on a white background. The relative levels of TJ5-13 and #1664 binding with each HA are shown to the left of each virus label as a heat map, ranging from white (no binding) to black (maximal binding). HA residues that are within the buried surface area of the interfaces with TJ5-13 and #1664 based on PDBePISA (32) are indicated. Residues comprising antigenic sites A and B are annotated, along with the status of glycosylation at position 158. (**C**) Cryo-EM maps and models at the interfaces of the light chain of each antibody in the vicinity of residue 158. Hydrogen bonds are shown by dashed lines, with those meeting the strict criteria in ChimeraX colored blue and those identified under more permissive criteria colored orange. (**D**) Interactions of the heavy chain of each antibody with COBRA HA NG2. The approximate locations of the close-up views within the overall structures are indicated by red boxes, with dashes indicating views that are obscured by elements in the foreground.

To assess the binding characteristics of TJ5-13 and #1664, we resolved the interface of each mAb with COBRA HA NG2 by Cryo-EM (Fig. 3; Fig. S4 to S6; Table 4). Comparing the two structures of TJ5-13:NG2 at 2.61 Å and #1664:NG2 at 3.45 Å, respectively, reveals that they adopt similar modes of binding to the NG2 head domain (Fig. 3A; Fig. S6). Both engage the HA predominantly via the heavy chain, which is positioned more proximal to the head domain compared to the light chain (Fig. 3A and B). Based on the models built into the cryo-EM maps, the relatively small region of the HA interface with the light chain includes residues 158–160 within antigenic site B (Fig. 3B and C). Interestingly, this region encodes for a glycosylation site in a subset of more recent HA sequences, including A/Switzerland/8060/2017, COBRA NG2, A/South Australia/34/2019, and A/Tasmania/503/2020. A close examination of the cryo-EM structures shows that this glycosylation site is resolved in both the TJ5-13 and #1664 complexes with NG2. While the glycan does not appear to contribute any direct interactions between the HA and the TJ5-13 antibody, the close proximity of Y37 in the #1664 light chain suggests the potential for transient hydrogen bonding. In both cases, however, the glycan adopts an orientation that points away from the binding interface. This suggests that the glycan can be readily accommodated by both antibodies, though the specific conformation imposed on the carbohydrate chain may impose an entropic cost to binding.

Beyond accommodation of this N-linked glycan, the light chains of both antibodies also form contacts with amino acids in this loop (Fig. 3C). In TJ5-13, this includes a hydrogen bond between the hydroxyl group of Y91 with the backbone of N158, as well as a hydrophobic surface formed by the side chains of Y91 and F96 that, in conjunction with residues on the heavy chain, form an ideal space for the aromatic ring of HA Y159. In #1664, this hydrophobic characteristic is also very prominent with Y37 and I38, likewise accommodating Y159. In addition, the N36 of #1664 is able to form hydrogen bond contacts with T160.

Looking more closely at the interface between the heavy chains of TJ5-13 and #1664 with NG2 reveals both similar and distinct traits in their modes of binding (Fig. 3D). Both engage the RBS with a mixture of polar and hydrophobic contacts. In the case of TJ5-13, the electrostatic interactions are quite pronounced with CDR loop 1 residues T30 and Y31a forming hydrogen bonds with D190 and Y98 of HA, respectively, CDR loop 2 residue N53 forming a potential hydrogen bond with S137, and CDR loop 3 residue N99 with HA S198. These are rounded out with hydrophobic interactions between F54 and Y31a within a hydrophobic patch formed by W153, T155, and L194. A similar array of electrostatic interactions can be observed for #1664 in the RBS, with the R110 in CDR loop 3 hydrogen bonding with the backbone of HA T135, the backbone of G111a interacting with S136, N111b interacting with both Y98 and the backbone of D190, and D36 in CDR loop 1 interacting with S137. A substantial portion of the interface within the RBS is formed by hydrophobic packing of residues in CDR loop 3, with G111a located in close proximity to I226, and P111c to W153 and L194. Along the edge of the RBS, F30 in CDR loop 1 packs near P227 and could potentially engage with R222 by cation-pi interactions, while the backbone of T29 forms a hydrogen bond with the same residue.

Beyond these central points of interaction, the heavy chains of both antibodies also form contacts with residues on the periphery of the RBS. In TJ5-13, amino acids in or near the CDR loop 3, including G96, A97, Y100c, and P100e, help accommodate the hydrophobic residues I192, F193, and Y159 in HA. These are further supported by polar interactions between the TJ5-13 residue N99 with HA S198 and the backbone of T28 with the side chain of K189. By comparison, the heavy chain of #1664 contains fewer points of direct contact outside the RBS, with the most notable regions being Y112b and F114 supporting a hydrophobic cleft for Y159. Intriguingly, E85 and other residues in the heavy chain, such as the backbone of S26 and Y28, also appear to form electrostatic interactions with the glycan tree of N165 in the neighboring HA protomer.

### Specific amino acids in antigenic sites A and B impact recognition by the TJ5-13 and #1664 antibodies

**Fig 4:**
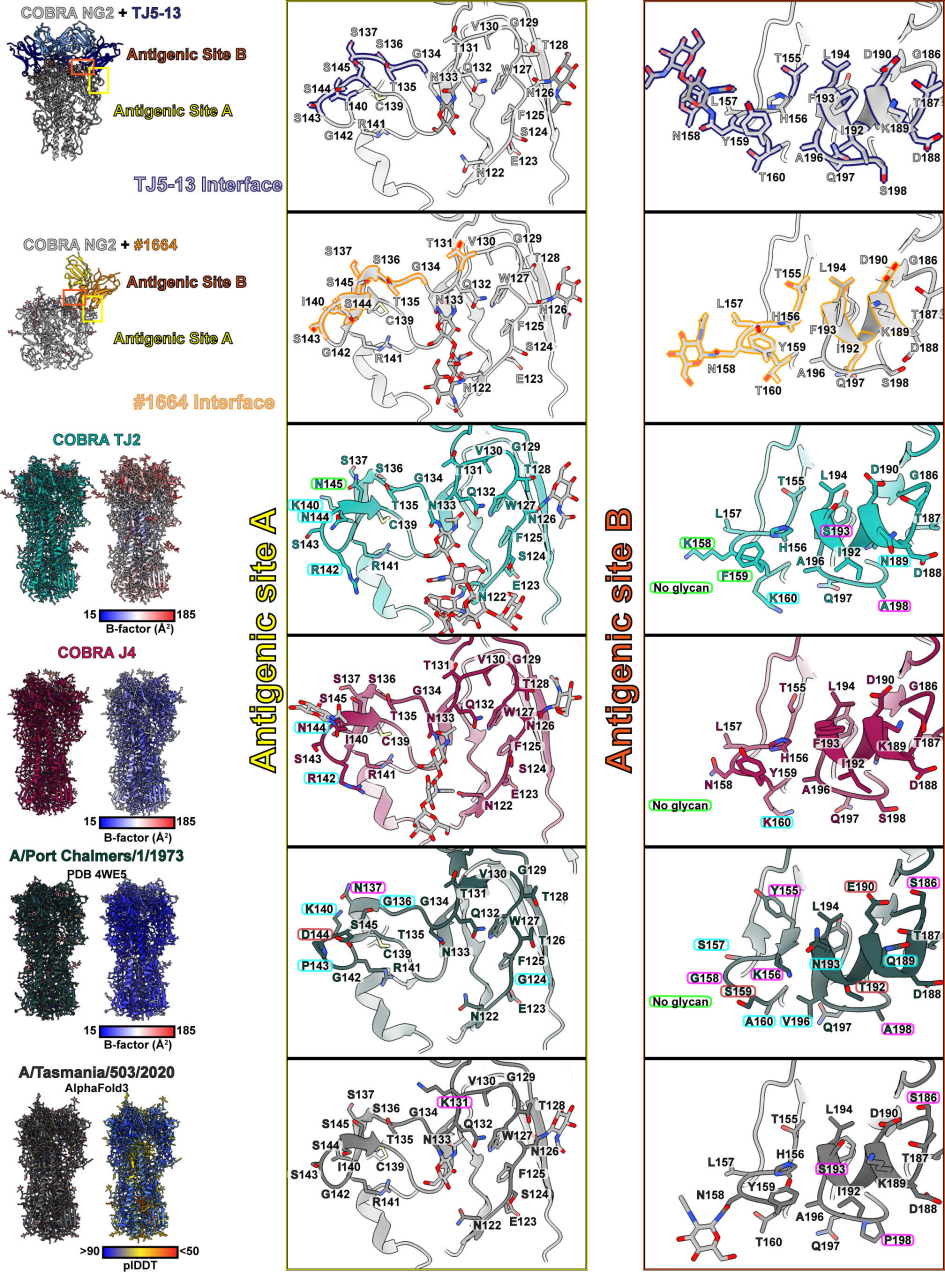
Comparison of antigenic sites A and B across divergent H3 HA proteins. COBRA HA NG2 residues that are involved in the interface with TJ5-13 and #1664 are outlined in dark blue and orange, respectively. Residue labels bordered in green indicate changes in an HA relative to COBRA HA NG2 that would likely improve binding, those bordered in light blue a weakly positive or neutral effect, magenta a neutral or slightly negative effect, and red a strong negative effect.

Of the HA proteins tested for binding with the TJ5-13 and #1664 antibodies, COBRA HA NG2 shows an intermediate affinity between the most potent and weakest binders. To investigate the molecular factors that may improve or worsen the recognition of HA by these antibodies, we performed structural comparisons of the NG2-Fab cryo-EM structures with representative HA proteins from the BLI assay panel. This included newly solved crystal structures of TJ2 and J4 as representative strong binders (Fig. 4; Fig. S7; Table 5), as well as a previously solved structure of the A/Port Chalmers/1/1973 HA (33) and an AlphaFold3 prediction of the A/Tasmania/503/2020 HA (34) to represent no binding and weak binding, respectively (Fig. 4; Fig. S8).

**TABLE 5:** Data collection and refinement statistics (molecular replacement)

	TJ2 COBRA (PDB 9B7H)	J4 COBRA (PDB 9B7I)
Data collection		
Space group	I2_1_	P1
Cell dimensions		
*a*, *b*, *c* (Å)	77.25, 117.68, 198.62	83.74, 105.30, 109.68
α, β, γ (°)	90.00, 90.95, 90.00	71.61, 73.38, 87.07
Resolution (Å)	45.66–3.15 (3.32–3.15)^*a*^	49.92–2.90 (2.95–2.90)^*a*^
Unique reflections	30,774 (4,475)	69,687 (3,369)
*R*_meas_	0.181 (1.651)	0.149 (0.449)
*I*/σ*I*	6.0 (0.9)	7.5 (1.0)
CC(1/2)	0.994 (0.578)	0.987 (0.898)
Completeness (%)	99.7 (99.8)	92.4 (89.0)
Redundancy	3.8 (3.9)	3.7 (3.6)
Wilson B-factor	96.19	56.00
Refinement		
Resolution (Å)	45.46–3.15 (3.26–3.15)	49.92–2.90 (3.00–2.90)
No. reflections (refinement)	30,733 (3,054)	69,518 (6,519)
*R*_work_/*R*_free_	0.222/0.269	0.228/0.286
No. atoms		
Protein	11,587	23,203
Ligand	852	1,125
Water	0	0
*B*-factors		
Average	107.22	66.92
Protein	104.78	65.78
Ligand	140.38	90.55
Water	NB^*b*^	NB
Ramachandran plot		
Favored (%)	97.67	95.98
Allowed (%)	2.33	4.02
Outliers (%)	0	0
R.m.s. deviations		
Bond lengths (Å)	0.003	0.003
Bond angles (°)	0.53	0.57

^*a*^ Values in parentheses are for the highest-resolution shell.

^*b*^ NB, no binding.

**Fig 5:**
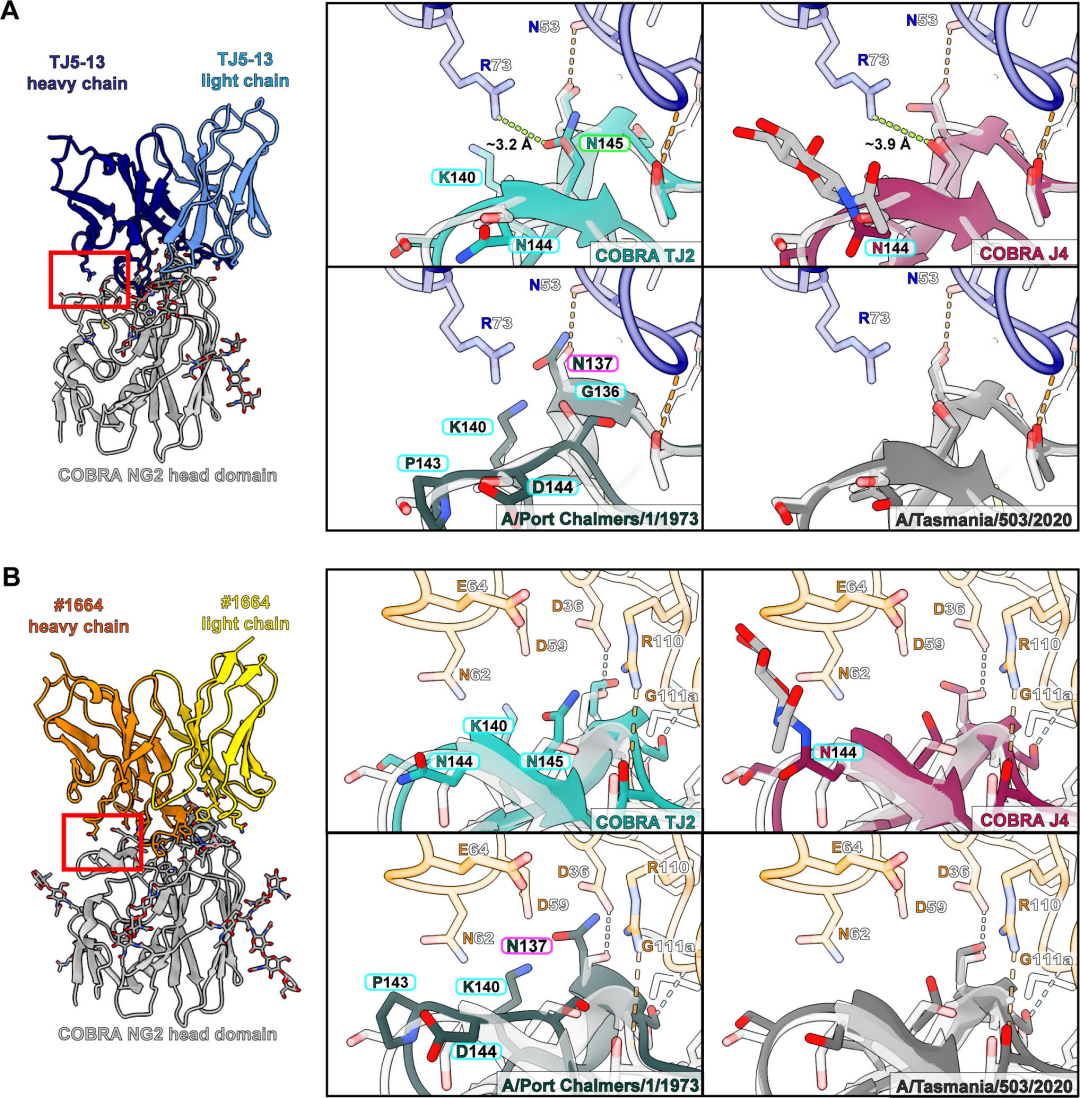
Potential interactions of antibodies TJ5-13 and #1664 with antigenic site A of different H3 HA proteins. (**A and B**) Overlays of the COBRA HA TJ2, COBRA HA J4, and A/Port Chalmers/1/1973 HA crystal structures and A/Tasmania/503/2020 HA AlphaFold3 model with the COBRA HA NG2:TJ5-13 complex (**A**) and COBRA HA NG2:#1664 complex (**B**). Hydrogen bonds are shown by dashed lines, with those meeting the strict criteria in ChimeraX colored blue and those identified under more permissive criteria colored orange. Green dashes indicate a potential electrostatic interaction with one of the other HA proteins. Residue differences that are expected to improve binding have labels bordered in green, those with a slightly beneficial or neutral effect in light blue, those with a neutral or slightly negative effect in magenta, and those with a strong negative effect in red.

A perusal of the HA regions within each antibody’s binding footprint revealed that antigenic sites A and B of HA show the greatest degree of impactful divergence between the strains (Fig. 4). In particular, specific amino acids in the 130-loop, 150-loop, and 190-helix show variable characteristics that can account for the relative improvement or reduction in binding. With antigenic site A, the interactions with TJ5-13 largely hinge on the ability of R73 and N53 in the heavy chain to form electrostatic interactions (Fig. 5A). In TJ2, the presence of an asparagine at residue 145 rather than a serine creates the potential for a new hydrogen bond with R73 by virtue of the shorter intermolecular distance. This feature is present in all the strains in the assay panel from 2005 to 2012, which correlates well with the most effective time period for this antibody. The COBRA HA J4, while lacking this same feature, shows some evidence in the electron density map of an N-linked glycan at residue 144 (Fig. S7B). Given the proximity, this may allow for additional interactions between the HA and the antibody via polar contacts. Interestingly, although the same glycan is not definitively observed in the COBRA HA TJ2 electron density map, the sequence of TJ2 possesses a canonical NXS motif in this same region that would have similar potential. This putative glycan is present in all the HA sequences from 1999 to 2013 apart from the A/Perth/16/2009 sequence, which encodes for K144 instead. While the HA sequence from A/Port Chalmers/1/1973 has several amino acid differences, their effect seems to be largely neutral, with accommodation of the larger N137 side chain by TJ5-13 N53 being the greatest potential hindrance to binding. When considering the interactions of #1664 with antigenic site A of HA, the situation is slightly different (Fig. 5B). Polar interactions are still involved via heavy chain residue R110. In this case, an asparagine residue at position 137 could present a steric hindrance, whereas an asparagine at position 145 could likely be accommodated. The proximity of #1664 E64, however, presents the possibility of additional interactions when a glycan is present at HA residue 144.

**Fig 6:**
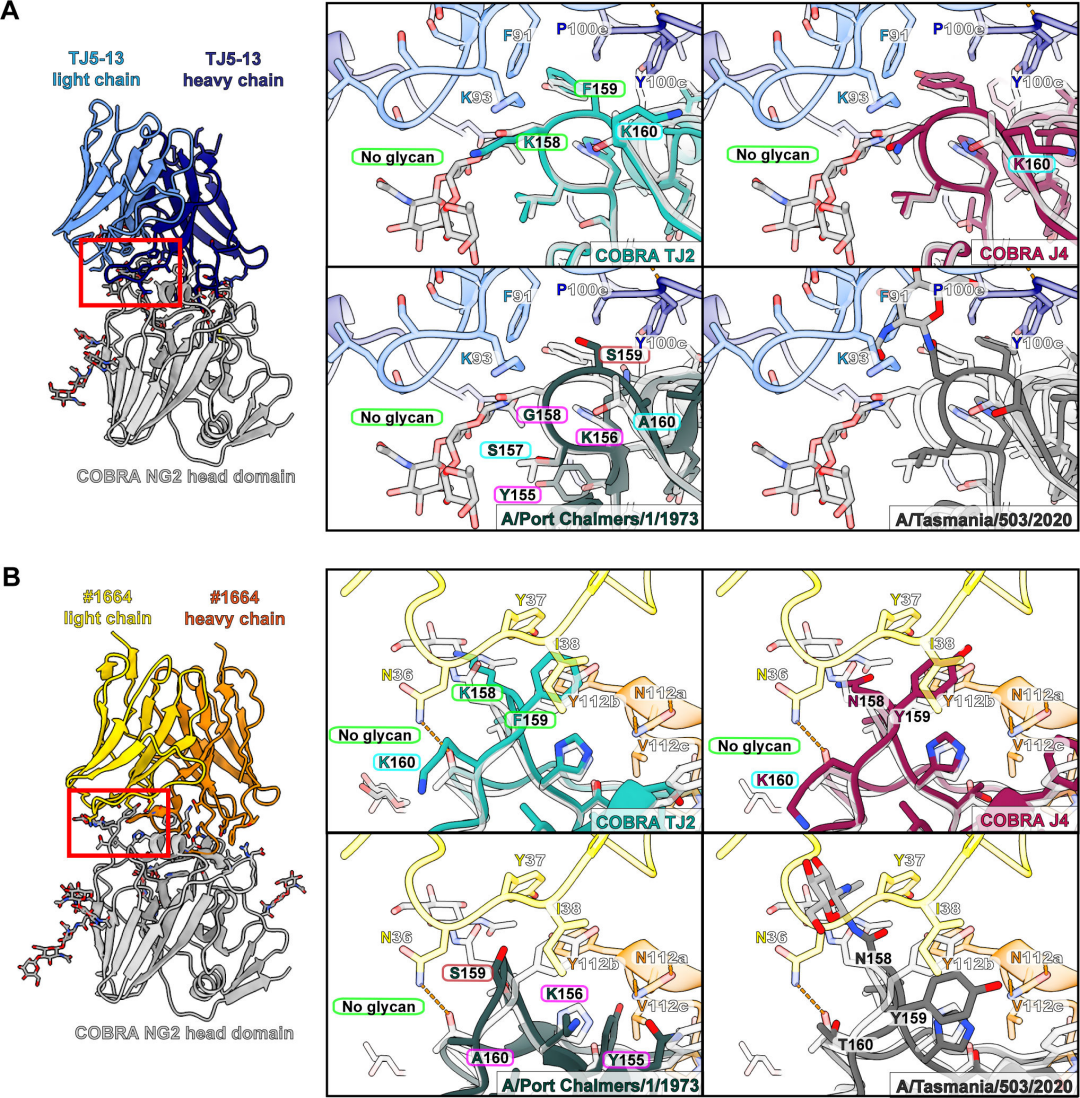
Potential interactions of antibodies TJ5-13 and #1664 with the 150-loop of antigenic site B of different H3 HA proteins. (**A and B**) Overlays of the COBRA HA TJ2, COBRA HA J4, and A/Port Chalmers/1/1973 HA crystal structures and A/Tasmania/503/2020 HA AlphaFold3 model with the COBRA HA NG2:TJ5-13 complex (**A**) and COBRA HA NG2:#1664 complex (**B**). Hydrogen bonds are shown by dashed lines, with those meeting the strict criteria in ChimeraX colored blue and those identified under more permissive criteria colored orange. Residue differences that are expected to improve binding have labels bordered in green, those with a slightly beneficial or neutral effect in light blue, those with a neutral or slightly negative effect in magenta, and those with a strong negative effect in red.

**Fig 7:**
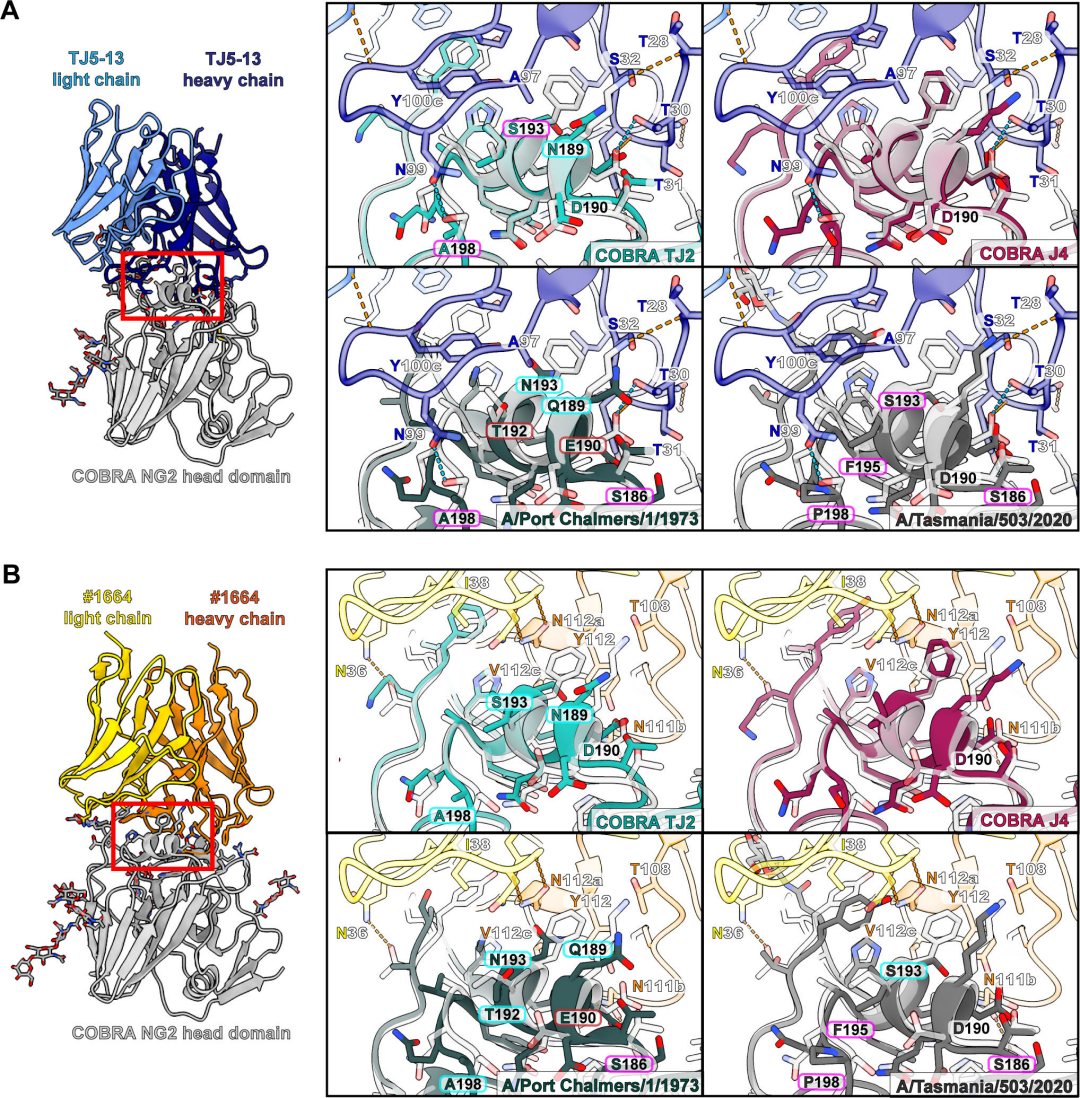
Potential interactions of antibodies TJ5-13 and #1664 with the 190-helix of antigenic site B of different H3 HA proteins. (**A and B**) Overlays of the COBRA HA TJ2, COBRA HA J4, and A/Port Chalmers/1/1973 HA crystal structures and A/Tasmania/503/2020 HA AlphaFold3 model with the COBRA HA NG2:TJ5-13 complex (**A**) and COBRA HA NG2:#1664 complex (**B**). Hydrogen bonds are shown by dashed lines, with those meeting the strict criteria in ChimeraX colored blue and those identified under more permissive criteria colored orange. Residue differences that are expected to improve binding have labels bordered in green, those with a slightly beneficial or neutral effect in light blue, those with a neutral or slightly negative effect in magenta, and those with a strong negative effect in red.

In the 150-loop of antigenic site B, the biggest factors influencing the binding interface are positions 158-160 in HA (Fig. 6). The presence of glycosylation at position 158 is correlated with a reduction in affinity in the BLI data for both antibodies, consistent with the steric restrictions imposed by the binding event. With position 159, both antibodies wrap this region with hydrophobic residues. In the case of HA sequences that encode a serine at this position, including A/Port Chalmers/1/1973, A/Kansas/14/2017, and A/Switzerland/9715293/2013, the binding is noticeably diminished. With TJ5-13, the impact is most pronounced when combined with a polar residue at position 193, suggesting synergy between these two positions (Fig. 6A and 7A). With #1664, position 159 alone is sufficient to influence binding independent of position 193. Even the difference in the absence or presence of an additional hydroxyl group, as seen with F159 in COBRA HA TJ2 versus Y159 in COBRA HA J4, is enough to alter the kinetics in binding via a faster dissociation rate (Fig. 6B; Fig. S2; Table 3). Beyond these central features of the 150-loop, additional residues may also influence the ability of these antibodies to bind HA. In particular, the larger side chains of Y155 and K156 in A/Port Chalmers/1/1973 may present additional steric hindrances at the binding interface.

In the 190-helix of antigenic site B, several residues play roles to varying degrees of importance depending on the antibody bound (Fig. 7). For TJ5-13, for example, the presence of D190 seems to be a key feature as the longer side chain of E190 in A/Port Chalmers/1/1973 would present a steric hindrance to binding. While this change could potentially impact #1664 in a similar way, there is likely more room for accommodating the longer side chain, albeit with restricted conformations. Beyond this, the antibodies diverge substantially in the way they engage with residues in the helix.

For TJ5-13, a strategic mix of hydrophobic and polar interactions appears to be the most optimal with HA residues 189, 192, 193, and 198 directly involved (Fig. 7A). COBRA HA J4, which is a perfect sequence match with COBRA HA NG2 in the 190-helix, contains the combination of K189 that can hydrogen bond with the main chain of TJ5-13, I192, and F193 which present an ideal hydrophobic surface for the interface, and S198 which would form a hydrogen bond with TJ5-13 N99. In COBRA HA TJ2, the lower hydrophobicity of S193 and loss of a hydrogen bond via A198 are partially mitigated by the retention of I192. In addition, N189, while not able to form the same hydrogen bond as K189, may still be able to interact electrostatically with residues in the local environment, such as TJ5-13 T30. By contrast, the combination of T192 and N193 in A/Port Chalmers/1/1973 essentially eliminates the potential for hydrophobic packing, while A198 lacks the ability to form a hydrogen bond. In the HA from A/Tasmania/503/2020, at first glance, the number of amino acid differences would not suggest the largely diminished binding based solely on direct contacts. Residues S186 and F195 are not directly in the binding interface, and S193 and P198 would be expected to have a similar effect as that seen for COBRA HA TJ2. Considered together, however, these changes suggest the potential for altered dynamics in the local structure of the helix. The change from glycine to serine at position 186 would reduce the flexibility of the loop leading into the helix, and P198 would make the loop after the helix more rigid. An additional factor is the change from tyrosine to phenylalanine at position 195, which would have subtle effects on the way this helix packs with other residues in the HA fold. Overall, these HA amino acid differences in A/Tasmania/503/2020 could make the local environment of the 190-helix more rigid and less amenable to induced-fit binding with TJ5-13.

With #1664, the 190-helix appears to play a minor role in determining affinity (Fig. 7B). The space is generally open around this helix, which allows accommodation of a range of residues, particularly at positions 192 and 193. Although polar residues such as serine or asparagine at position 193 appear in principle to be more favorable compared to a hydrophobic residue like phenylalanine, the impact seems to be much lower compared with drivers of binding in other parts of the interface.

In summary, the ability of TJ5-13 and #1664 to recognize divergent strains of H3N2 HA proteins depends on specific amino acids in antigenic sites A and B of HA. Antigenic site B appears to have the most impact on these interactions. The structural differences in different strains have varying degrees of impact. The 150-loop is a common factor for both antibodies, as a glycosylation site in this loop correlates with a reduction in the association rates. Residue 159 is also impactful in these interactions and seems to be the most important residue for influencing binding by #1664. For the rest of the antigenic site B, the 190-helix has a greater impact on binding by TJ5-13, and the composition of the 190-helix can have synergistic effects with the 150-loop for this antibody. For both antibodies, antigenic site A plays a peripheral role that can strengthen or weaken the binding in the context of the overall binding interactions.

## DISCUSSION

One of the primary goals of next-generation vaccine designs is to induce, or recall, potent broadly neutralizing antibodies. Several designs in development have shown encouraging results on this front, including the COBRA design strategy, which uses a layered consensus sequence approach to incorporate strongly conserved features into an antigen sequence. As these new vaccine designs are developed, there is a need to consider how they will induce or recall broadly neutralizing antibodies and how viruses might respond to increasing selective pressure from these antibodies. This latter factor is especially critical in considering the durability of broadly protective antibody responses and how this impacts the need to formulate or re-formulate next-generation vaccines. Toward this end, we characterized how three broadly neutralizing antibodies isolated from individuals vaccinated with Fluzone containing A/Hong Kong/4801/2014 as the H3 component (25, 26) performed against a wide array of HA proteins from historical and post-vaccine drifted strains as well as COBRA HA proteins.

Analysis of the TJ5-1 antibody revealed that it strongly binds a highly conserved epitope in the HA stem region. This included part of the fusion peptide, consistent with the ability of TJ5-1 to inhibit proteolytic activation of the HA by trypsin. Only a single HA amino acid difference in the interface had a substantial impact on binding, with that occurring in the oldest historical strain tested, A/Port Chalmers/1/1973. Since 1999, the surface targeted by this antibody has remained largely unchanged. Given the momentum of stem-focused vaccine designs in clinical development, including chimeric HA (13) and stem-presenting nanoparticles (12), this lack of substantial antigenic drift stands as a potential encouraging sign for these vaccine strategies. Whether this would remain the case under the more stringent selective pressure applied by these vaccines merits more investigation, as escape mutants in the HA stem have already been observed for H1N1 viruses (35). At the outset, however, the temporal breadth of TJ5-1 presents a point of optimism on the potential of H3N2 stem-focused vaccines for durable protection. This is reinforced when comparing TJ5-1 with the other structurally characterized mAbs that target the H3 stem, CR8020 and CR8043. Because of the difference in binding orientations of these two antibodies, there is a difference in the relative sensitivities to known escape mutants (30). The CR8020 and CR8043 mAbs specifically are affected differently by the mutations D348N, R354M, G362E, and Q363T (HA2 numbering D19N, R25M, G33E, and Q34T). The shifted orientation of TJ5-1 would bring its distinct sensitivities to these known sites of antigenic variation. Furthermore, although TJ5-1, CR8020, and CR8043 bind overlapping epitopes, the VH chain usage for all three is different. CR8020 and CR8043 utilize the germline genes *V_H_1-18* and *V_H_1-3*, respectively (30), whereas TJ5-1 uses *V_H_1-2* (Table 1). In the human heavy chain repertoire, each of these genes is estimated to be present in 2%–6% of circulating antibodies (36). This presents the potential for diverse polyclonal antibody responses to the H3 stem that are refractory to some forms of antigenic drift.

With the TJ5-13 and #1664 antibodies, the situation is more complex. Both demonstrate broadly neutralizing activity by virtue of targeting the RBS, but clearly demonstrate limitations from antigenic drift. The most optimal time period for both of these antibodies corresponds to strains from 2005 to 2012. With the exception of the HA protein from A/Port Chalmers/1/1973, however, all of the HA proteins showed a measurable degree of binding through affinity or avidity effects. This suggests that single-point mutations rarely oblate the effectiveness of these antibodies. Rather, a reduction in efficacy is typically rooted in an accumulation of changes over several seasons. At the same time, some variable regions in HA have more weight than others. This is most clearly seen from changes in the 150-loop. The introduction of N-linked glycosylation at residue 158 is a major factor in reducing the affinity for these antibodies in some strains after 2014. Interestingly, HA sequences from A/Hong Kong/4801/2014 and A/Singapore/INFIMH-16-009/2016 both contained this NXT glycosylation site in the original isolates but lost it during egg adaptation due to a T160K mutation (37–39). For split inactivated virion vaccines generated from eggs, this raises an important issue of antigenic mismatch due to changes in the virus during vaccine production. This would be consistent with the A/Hong Kong/4801/2014 component in the egg-derived seasonal vaccine, effectively recalling the TJ5-13- and #1664-like antibodies that work effectively for historic strains but less well for contemporary and future strains. In these circumstances, recombinant subunit vaccines may present an advantage due to the ability to strictly control the sequence composition of the vaccine product without being subject to the constraints of growing whole virus to high titer. Beyond the glycosylation site, residue 159 of HA has the largest impact on binding by these antibodies, particularly for #1664, with other variable residues in antigenic sites A and B serving more peripheral roles.

Interestingly, the amino acid sites associated with variable binding by these broadly neutralizing antibodies correspond to several known residue “hotspots” that drive antigenic drift in H3N2 viruses (25, 40–43). This is particularly true of residues in the 150-loop and 190-helix in antigenic site B. Of these well-documented residues, amino acids involved in the presence/absence of glycosylation at position 158 and the amino acid present at position 159 are particularly dynamic in the mutational landscape of recent H3N2 strains (40–42). Intriguingly, after the 158-glycosylation site emerged in 2014, by the 2021–2022 season, it reverted to a non-glycosylated state in the dominant strains circulating in the northern hemisphere (40, 41). These changes have corresponded with other substitutions, particularly those changing the hydrophobicity at position 159, that are driving recent patterns of antibody escape. The impact of these residues on TJ5-13 and #1664 suggests that the general patterns of H3N2 antigenic drift are recapitulated at a smaller scale with some RBS-targeting broadly neutralizing antibodies. This may present a challenge to attaining universal protection by head-targeting antibodies, as even very robust RBS-targeting antibodies may be subject to diminishing effectiveness over time. For next-generation vaccines that incorporate the H3 head domain, it may be the case that re-formulation will still be needed periodically to keep up with antigenic change. One advantage of utilizing COBRA HAs is the capacity to rapidly design antigens that represent antigenically distinct clades of viruses without being biased by any one strain, potentially eliciting a greater breadth of antibody responses that decrease season-to-season variability in vaccine efficacy.

This study has several inherent limitations. While the antibodies assessed were isolated from human subjects, they do not necessarily capture the entire, complex dynamic of a polyclonal antibody response. Because immune responses to influenza are also heavily influenced by exposure history, generational gaps can exist in which the same mutation at a given residue can have either escape or sensitizing properties depending on which subset of the human population is sampled (44). As a result, these specific antibodies do not necessarily represent the strengths or shortcomings of broadly neutralizing antibodies more generally. It is also not clear at what point a reduction in *in vitro* affinity translates to a reduction of efficacy *in vivo*. Taken with this context in mind, however, it provides specific insights into the molecular mechanisms that influence the robustness of broadly neutralizing antibodies against antigenic changes in H3N2 viruses over time.

In conclusion, broadly neutralizing antibodies against H3 show varying degrees of resilience to antigenic drift. An epitope targeted by a stem-directed antibody shows little change over a half-century timespan, suggesting the potential for long-term efficacy of this antibody. Two antibodies directed toward the RBS show strong patterns across more than a decade but show specific susceptibilities to amino acid substitutions and glycosylation patterns in antigenic sites A and B. This has important implications for the design, selection, and implementation of next-generation vaccine candidates, including how production platforms impact antigenic characteristics and the active trends of H3N2 virus evolution.

## MATERIALS AND METHODS

### Biolayer interferometry of antibodies TJ5-1, TJ5-13, and #1664 with recombinant HA

Recombinant soluble HA proteins (rHA) were expressed and purified from mammalian cell culture by the Center for Influenza Research for High-Risk Populations (CIVR-HRP) Assay and Reagent Core as previously described (45). Accession codes for the source sequences of strain-specific HA proteins are listed in Table 6. Briefly, coding sequences corresponding to the ectodomain of each H3 HA protein fused to a C-terminal T4 fibritin trimerization (“Foldon”) domain, Avi-Tag, and 6× His-tag were cloned into the pcDNA3.1/Zeo (+) vector. Expi293F cells were transiently transfected, and the secreted rHA was purified by immobilized metal ion affinity chromatography. The HC and LC of mAb #1664, originally discovered from a human cohort receiving the quadrivalent vaccine during the 2016–2017 northern hemisphere influenza season (26), were synthesized and cloned in the pcDNA3.4 TOPO vector (catalog no. A14697, Thermo Fisher Scientific) by GenScript (Piscataway, NJ) and expressed in EXPI293 cells. Mab purification was performed using the conditioned supernatants of transfected cells and quantified, as previously described (46). In brief, mAb #1664 was purified by affinity chromatography using HiTrap protein G HP columns (catalog no. 17040501, Cytiva). The conditioned supernatant was applied to protein G columns and purified through the AKTA pure system (Cytiva). The mAbs were eluted using 0.1 M glycine [pH 2.5] (MilliporeSigma). The eluted protein was immediately neutralized with 200 µL of 1.5 M Tris (pH 8.8) (MilliporeSigma), and the protein-containing elution fractions were pooled, buffer-exchanged into PBS containing 0.05% sodium azide (MilliporeSigma), and concentrated using an Amicon Ultra-15 centrifugal filter unit (catalog no. UFC905024, MilliporeSigma). The concentrations of purified mAb were determined using a micro BCA (bicinchoninic acid) assay kit (catalog no. 23235, Thermo Fisher Scientific) using a human IgG standard (catalog no. 401114, MilliporeSigma), and purity was assessed by SDS-PAGE and Western blot (Thermo Fisher Scientific). TJ5-1 and TJ5-13 were identified from screening of expanded B cells after vaccination of subjects with the 2017–2018 quadrivalent influenza vaccine against a panel of H3 HAs, including that of A/Hong Kong/4801/2014 and COBRA HAs TJ5, J4, and NG2, as previously described (25). Briefly, B cells expressing HA-reactive mAbs were selected for electrofusion with a myeloma cell line to generate hybridomas. Hybridomas were subjected to single-cell sorting and subsequent rounds of screening against the original HA panel to downselect individual clones with the highest binding signal, from which those corresponding to TJ5-1 and TJ5-13 were isolated. TJ5-1 and TJ5-13 hybridomas were grown in 250 mL of serum-free medium (Gibco) for approximately 1 month. To purify mAbs, culture supernatants were run through protein G columns (GE Healthcare), eluted using 0.1 M glycine (pH 2.7), and neutralized immediately with 1 M Tris (pH 8.0), buffer-exchanged into PBS, and concentrated for use in downstream assays.

**TABLE 6:** Recombinant HA accession numbers^*a*^

Protein sequence	GISAID accession
A/Tasmania/503/2020 HA	EPI_ISL_483574
A/South Australia/34/2019 HA	EPI_ISL_395032
A/Switzerland/8060/2017 HA	EPI_ISL_303951
A/Kansas/14/2017 HA	EPI_ISL_403059
A/Singapore/INFIMH-16-0019/2016 HA	EPI_ISL_239803
A/Hong Kong/4801/2014 HA	EPI_ISL_16706634
A/Switzerland/9715293/2013 HA	EPI_ISL_162149
A/Texas/50/2012 HA	EPI_ISL_129858
A/Victoria/361/2011 HA	EPI_ISL_118588
A/Perth/16/2009 HA	EPI_ISL_167306
A/Brisbane/10/2007 HA	EPI_ISL_176548
A/Wisconsin/67/2005 HA	EPI_ISL_115646
A/Panama/2007/1999 HA	EPI_ISL_111375
A/Port Chalmers/1/1973 HA	EPI_ISL_123257

^*a*^ Sequences can be found at https://gisaid.org/.

Biolayer interferometry experiments were performed using an Octet RED384 instrument (Sartorius). Exploratory assays were performed using 1–5 μg/mL of antibody for loading onto anti-hIgG Fc Capture (AHC) biosensors and 100 nM of each rHA as the analyte. The binding characteristics of these initial assays were used to select concentration ranges for the dilution series of the rHA with each antibody and optimize the timing of each assay step. For the final assays, each antibody was loaded at 1 µg/mL and tested against three concentrations along a threefold dilution series of each rHA, all diluted in Assay Buffer (phosphate-buffered saline pH 7.4 [PBS; Sigma Aldrich], 1% bovine serum albumin [BSA; Fisher Scientific], and 0.05% Tween [Fisher Scientific]). Samples and reagents were all loaded into tilted 384-well low protein binding plates (Sartorius). Pre-hydrated AHC biosensors were first dipped into Assay Buffer for 10 min, followed by loading to a wavelength shift threshold value of 0.5 nm for all biosensors. The biosensors were returned to the Assay Buffer for 2 min to measure a baseline, transferred into the sample wells to perform Association for 10 min, then dipped back in the same Assay Buffer for 20 min to assess Dissociation. Biosensors loaded with antibody but dipped into Assay Buffer during the Association step were included for use in baseline subtraction. All assays were performed with two technical replicates. The data were analyzed in the Octet Data Analysis HT software v7 (Sartorius). Savitzky-Golay filtering was applied to remove high-frequency noise and corrections were applied to account for baseline drift, normalize the Y-axis, and reconcile inter-step jumps in the signal. To account for the presence of multiple binding sites on the rHA, a bivalent analyte (1:2) model was utilized for curve fitting in kinetic analysis. Due to the slow dissociation rates of the interactions, in cases where sensor-to-sensor variability introduced problems with the data (no K_D_ calculated or replicates different by more than one order of magnitude), the impacted replicate groups were repeated. Final kinetic values are derived from the group fitting the curves within each replicate (Supplemental Data, Dryad). The processed curves with their associated model fits are presented in Fig. S2.

### Expression and purification of proteins for structural analyses

TJ5-13 fragment antibody-binding region (Fab) was prepared either by digestion of a full-length monoclonal antibody (mAb) or by expression of a His-tagged Fab. For full-length antibody expression, variable (Fv) regions of TJ5-13 were synthesized and cloned into a vector encoding a CMV promoter with a chimeric beta globin intron and flanking WPRE sequence as IgG1 heavy chain and lambda light chain expression plasmids (Twist Biosciences). Protein expression was performed by transient transfection of suspension-adapted Chinese Hamster Ovary Cells (CHO-S) purchased from Thermo Fisher. Prior to transfection, cells were maintained at a density of 0.3–2.0 million cells/mL in CD-CHO medium supplemented with 8 mM GlutaMax and 1× Hypoxanthine/Thymidine in shake flask cultures at 37°C, 8% CO_2_, 85% humidity at 125 rpm in an ISF1-X shaker incubator (Kuhner, Birsfelden, Switzerland). The cells were transfected using an STX electroporation device (MaxCyte Inc., Gaithersburg, MD) according to the manufacturer’s protocols. For protein production, the growth medium was adjusted to CD OptiCHO supplemented with 0.1% pluronic acid, 2 mM GlutaMax, and 1× H/T and 1 mM sodium butyrate, supplemented with CHO Growth A, 0.5% Yeastolate (BD, Franklin Lakes, NJ, US), 2.5% CHO-CD Efficient Feed A, and 0.25 mM GlutaMax, 2 g/L Glucose (Sigma-Aldrich St. Louis, MO, US). Cells were maintained at 32°C, at a concentration of 1.0 million cells/mL, until cell viability dropped to below 50% (usually following 8–14 days in culture). All media and supplements were purchased from Thermo Fisher, Life Technologies, Carlsbad, CA, unless stated otherwise. Monoclonal antibody was purified from the culture supernatant using Protein A Sepharose (Cytiva) with commercially available IgG Binding and IgG Elution buffers (Thermo Fisher) using an AKTA purification system. Fractions from the low pH elution were neutralized using 1 M Tris (pH 8.5), and the mAb was digested with papain (Pierce Fab Preparation Kit, Thermo Scientific). The recovered Fab was polished by size exclusion chromatography on a Superdex 200 10/300 column (Cytiva) equilibrated in PBS.

Direct expression and purification of TJ5-13 Fab was accomplished by transient transfection of CHO-S cells (as described above) with the TJ5-13 light chain plasmid plus a Fab heavy chain (comprised of TJ5-13 VH and CH1 domains) containing a 6xHis tag and an upstream thrombin cleavage site. Culture supernatant was harvested 8 days after transfection, and Fab was recovered by Ni-NTA affinity chromatography. Briefly, the recovered supernatant was diluted with an equal volume of a buffer containing 500 mM NaCl, 20 mM sodium phosphate (pH 8), 20 mM imidazole, and loaded onto a Ni-NTA HisTrap Crude FF column (Cytiva) on an AKTA system. The column was washed with the same buffer, and then the protein was eluted with a linear gradient to a buffer containing 500 mM NaCl, 20 mM sodium phosphate (pH 8), 500 mM imidazole. Final sample polishing was achieved by size exclusion chromatography step using a Superdex 200 10/300 column equilibrated in 150 mM NaCl, 50 mM Tris (pH 8).

The COBRA HA NG2 protein was similarly expressed in CHO-S cells using a CMV-driven vector derived from pcDNA3.1 (47). The cultures were harvested 5 days after transfection, and His-tagged NG2 (NG2^CHO^) was purified from the supernatant with Ni-NTA as described above prior to polishing on a Superdex 200 10/300 column (Cytiva) equilibrated in 150 mM NaCl, 50 mM Tris (pH 8). The purified sample was concentrated, then flash-frozen using liquid nitrogen and stored at −80°C until use.

To generate TJ5-1 Fab and #1664 Fab for determination of the TJ5-1:NG2 and #1664:Fab structures, the TJ5-1 mAb was purified from hybridoma culture media or the #1664 mAb from transfected 293 cells using a Protein G column (Cytiva). The TJ5-1 mAb and the #1664 mAb were then digested into their Fab fragments using the Pierce Fab Purification Kit (Thermo Fisher Scientific). The Fab fragments were isolated from the Fab digestion mixture using a HiTrap MabSelect column (Cytiva). A twofold excess of TJ5-1 or #1664 Fab was then added to Expi293F-expressed NG2 in 20 mM Tris (pH 7.5), 100 mM NaCl, and incubated at 4°C overnight. The mixture was then subjected to size exclusion chromatography (SEC) on a Superdex 200 Increase 10/300 Gl column (GE Healthcare) to isolate the Fab:NG2 complex from excess Fab.

### Single-particle cryo-electron microscopy of rHA-Fab complexes

Preliminary data for the TJ5-13 Fab bound to NG2 were generated using Fab fragments derived from papain digestion combined with NG2^CHO^ in a 3:1 ratio. The sample concentration was adjusted to 0.5 mg/mL with PBS and supplemented with 5 µM lauryl maltose neopentyl glycol (LMNG) prior to depositing on glow-discharged Quantifoil R1.2/1.3 400 mesh grids. Blotting and plunge freezing in liquid ethane were performed with a Vitrobot Mark IV (ThermoFisher Scientific) at room temperature and 100% humidity. The sample was screened and collected on a ThermoFisher Scientific Glacios cryo-TEM at an accelerating voltage of 200 kV coupled to a Gatan K2 Summit direct electron detector and a pixel size of 0.6915 Å/pixel located at the UCSC Biomolecular cryo-EM facility. The data were collected in SerialEM (48, 49) in counting mode at a nominal magnification of 57,000×, exposure time of 5 s, and a total dose of ~50 electrons/Å^2^. The data collected (4,044 movies) were processed in CryoSPARC v3.2 (50) with motion and CTF correction performed using PatchMotion (51) and PatchCTF, respectively. The curation of the best movies according to CTF, motion distance, and astigmatism resulted in 3,954 movies that were used in the following steps. Unbiased particle picking was performed using the Blob Picker, followed by curation and particle extraction using a box size of 588 pixels. The initial 886,314 particles were submitted to multiple rounds of 2D classification to clean the data set and select the best classes. The top 59,358 particles were used to generate three initial volumes by *ab initio* reconstruction. The best volume and top 30,733 selected particles were subjected to multiple rounds of homogeneous and non-uniform refinements (52), ultimately yielding a ~ 5 Å volume with C3 symmetry imposed.

For high-resolution data collection, TJ5-13 Fab expressed with a His-tag was combined in molar excess with COBRA NG2^CHO^ and incubated at 4°C for 24 h. Subsequently, the complex was purified, and the complex was isolated by size exclusion chromatography using a Superose 6 10/300 Gl column (Cytiva) equilibrated with 150 mM NaCl, 50 mM Tris (pH 8). Fractions were pooled based on the chromatogram and SDS-PAGE analysis, then concentrated to 1.1 mg/mL. Sample was applied to glow-discharged Quantifoil R1.2/1.3 400 mesh grids at concentrations of 0.5 or 1 mg/mL, with or without supplementation with 5 µM LMNG, then blotted and plunged in liquid ethane using a Vitrobot Mark IV (ThermoFisher Scientific) at room temperature and 100% humidity. Grids were screened for quality at the UCSC cryo-EM facility prior to storage in liquid N_2_. Data collection on two grids prepared with 1 mg/mL sample, one with and one without LMNG, was performed on a Titan Krios G2 cryo-TEM coupled with a Gatan K3 direct electron detector at an accelerating voltage of 300 kV located at the W.M. Keck Foundation Advanced Microscopy Laboratory—UCSF. The data were collected in SerialEM (48, 49) in counting mode at a nominal magnification of 105,500×, pixel size of 0.835 Å/pixel, exposure time of 2 s, and a total dose of 45.8 electrons/Å^2^. The data from each were combined for a total of 13,223 movies and processed together using CryoSPARC v3.2 and v4.2.1 (50). Motion correction and CTF estimation were performed with PatchMotion and PatchCTF (51). The top 12,851 curated movies were used in the following steps. The 3D volume of the ~5 Å preliminary structure was smoothed by Gaussian filtering and then used to generate 2D templates for particle picking, followed by curation and particle extraction using a box size of 480 pixels. An initial 3,186,155 particles were classified by multiple rounds of 2D classification prior to generating *ab initio* reconstructions. The best 298,059 selected particles were then used to generate three initial 3D reconstructions for the following 3D classification.

The best volume and top 70,400 particles were further processed by homogeneous and non-uniform refinements (52) and local CTF refinements prior to the estimation of local resolution. The final map was sharpened in CryoSPARC based on a B-factor of 63.3 Å^2^ with a “gold-standard” FSC_0.143_ nominal resolution of 2.61 Å. A homology model for NG2 was generated by SwissModel (53) and docked into the volume using UCSF Chimera. A model for the Fv region of TJ5-13 was generated with ABodyBuilder (54) and placed in the map using Coot version 0.9.8.92 (55). Further modeling, coordinate refinement, and energy minimization were performed using Coot, ISOLDE version 1.6.0, and Phenix version 1.20.1-4487 (55–57). Validation of the model was performed using MolProbity (58) for protein geometry and Privateer in the CCP-EM 1.7.0-rc interface (59–61) for glycan structure. The maps and model were deposited in the Electron Microscopy Databank and Protein Data Bank, respectively, with accession numbers EMD-44305 and PDB ID 9B7G. Data collection and model refinement parameters are reported in Table 4. Figures were generated using UCSF ChimeraX (7).

The TJ5-1 Fab bound to NG2 and the #1664 Fab bound to NG2 complexes, purified by SEC, were applied to glow-discharged carbon/copper grids for grid plunging and data collection. The NG2:TJ5-1 data were collected on a Titan Krios cryo-TEM coupled with a Gatan K2 Summit direct electron detector at an accelerating voltage of 300 kV. The data were collected in counting mode at a nominal magnification of 22,500, pixel size of 1.093 Å/pixel, and a total dose of 60.2 electrons/Å^2^. A total of 5,440 movies were imported to CryoSPARC v4.3.1 and processed for Patch Motion Correction, Patch CTF estimation, particle picking, and two-dimensional class averaging. Following successive rounds of class averaging, 1,039,511 selected particles were used for *ab initio* reconstruction (10 classes, C1 symmetry) and heterogeneous refinements (10 classes, C3 symmetry). After assessment, the top *ab initio* class and its associated particles (248,800) were further processed by non-uniform refinement with C3 symmetry imposed. The final map was sharpened based on a B-factor value of 112.6 Å^2^ and had a “gold standard” FSC_0.143_ nominal resolution of 3.24 Å. The maps and model were deposited in the Electron Microscopy Databank and Protein Data Bank, respectively, with accession numbers EMD-47024 and PDB ID 9DN2. Data collection and model refinement parameters are reported in Table 4. Figures were generated using UCSF ChimeraX.

The NG2:#1664 data were collected on a Glacios coupled with a ThermoFisher Falcon IV direct electron detector at an accelerating voltage of 200 kV. The data were collected in counting mode at a nominal magnification of 190,000, pixel size of 0.526 Å/pixel, and a total dose of 57.0 electrons/Å^2^. A total of 20,527 movies were imported into CryoSPARC v4.3.1 and processed for Patch Motion Correction, Patch CTF estimation, particle picking, and two-dimensional class averaging. Following successive rounds of class averaging, 110,663 selected particles were used for *ab initio* reconstruction (C1 symmetry, single class), followed by non-uniform refinement in C1 symmetry. This revealed partial occupancy of the Fab, with only one of three potential binding sites possessing a strong signal. Particle subtraction followed by masked local refinement (C1 symmetry) was performed to focus on the HA head domain trimer with a single Fab. The final locally refined map, with a “gold standard” FSC_0.143_ nominal resolution of 3.45 Å, was sharpened based on a B-factor of 90.4 Å^2^. The handedness of the map was subsequently flipped in ChimeraX to enable model building. The refined maps of NG2:TJ5-1 and NG2:#1664 were then used for model building in Coot, followed by refinement in Phenix (55, 57). The maps and model were deposited in the Electron Microscopy Databank and Protein Data Bank, respectively, with accession numbers EMD-47071 and PDB ID 9DO2. Data collection and model refinement parameters are reported in Table 4. Figures were generated using UCSF ChimeraX.

### Trypsin cleavage inhibition assay

Stem-binding mAb TJ5-1 was assessed for its ability to prevent proteolytic cleavage and maturation of the NG2 HA0 precursor protein into the HA1/HA2 subunits using an adapted protocol (62). 40 µg of the mAb or 40 µL of PBS was added to 4 µg of the NG2 HA0 protein. The mAb:NG2 mixture was then incubated at 37°C for 1 h, then a 1:1 vol ratio of PBS (for the no mAb control) or TPCK-trypsin at 5 µg/mL (for the mAb treatment) was added. The mixture was then either not incubated for the untreated control or incubated at 37°C for 5, 20, 40, or 60 min. The sample was then run under reducing conditions on SDS-PAGE and stained with Coomassie blue to visualize the extent of NG2 HA0 cleavage. The NG2 HA0 band was expected at ~100 kDa, ~70 kDa for the HA1 subunit, and ~30 kDa for the HA2 subunit. Inhibition of proteolytic cleavage was assessed as the retention of the ~100 kDa NG2 HA0 band at all time points, whereas active cleavage was visualized as a decrease in HA0 band density across the time points.

### Expression, purification, and crystallization of TJ2 and J4 COBRAs

A gene fragment corresponding to the COBRA HA TJ2 ectodomain (residues 7–507) was codon optimized for insect cells and synthesized in frame with an N-terminal gp67 signal peptide and C-terminal thrombin cleavage site, Foldon domain, His-tag, and StrepTag, then cloned into the pBacPAK8 vector (GenScript). A gene fragment corresponding to the COBRA HA J4 (residues 7–506) was PCR amplified from a mammalian expression plasmid and cloned in-frame with the same elements in pBacPAK8 by Gibson Assembly (63) with a pre-formulated Master Mix (New England Biolabs). Both plasmids were used to generate baculovirus stocks using the *flash*BAC system (Mirus Bio). Proteins were expressed by adding 20–25 mL of amplified virus stock to 1 L of Sf9 cells (density of 2–4 million cells/mL) and incubating at 28°C, shaking at 120 rpm, for 3 days. The cells were pelleted by centrifugation and the supernatant recovered, buffered with concentrated NaCl and Tris (pH 8) to final concentrations of approximately 150 mM NaCl and 50 mM Tris, and stored at −20°C until further use. For purification, the frozen samples were thawed at 4°C for 24-48 hours, then filtered sequentially through class microfiber, 0.45 µm, and 0.22 µm filters to remove precipitates and aggregates. The samples were subsequently concentrated at room temperature by tangential flow using VivaFlow 200 cassettes with a nominal 30,000 molecular weight cutoff (Sartorius). The concentrated sample was mixed with an approximately equal volume of Buffer A (500 mM NaCl, 20 mM sodium phosphate [pH 7.4], 20 mM imidazole) and re-filtered using a 0.22 μm vacuum filter. The protein was loaded at room temperature onto a 5 mL Ni-NTA HisTrap column (GE Life Sciences) pre-equilibrated with Buffer A. The column was subsequently washed with Buffer A before transferring to an AKTA system at 4°C, where the protein was eluted by transitioning with a linear gradient to Buffer B (500 mM NaCl, 20 mM sodium phosphate [pH 7.4], 500 mM imidazole). Fractions were pooled based on the chromatogram and stored at 4°C until further use.

To prepare the samples for crystallography, both proteins were dialyzed in a buffer containing 150 mM NaCl, 20 mM Tris (pH 7.5), 2.5 mM CaCl_2_ overnight. The samples were then removed from dialysis and incubated with thrombin at 4°C overnight. Cleavage was assessed by SDS-PAGE prior to concentrating the samples and further purification using a Superdex 200 10/300 column (GE Life Sciences) equilibrated in Buffer C (50 mM NaCl, 10 mM Tris [pH 7.5]). The fractions were assessed for purity by SDS-PAGE prior to pooling and concentrating for crystallization attempts. Both proteins were screened using a Crystal Gryphon Robot (Art Robbins Instruments) with conditions from the MCSG suite (Anatrace) at concentrations of 9.7 mg/mL and 9.3 mg/mL, respectively, for TJ2 and J4. Initial hits appeared in days to weeks and were reproduced manually for optimization along salt and precipitant gradients. Crystals initially produced from these efforts failed to diffract to high resolution. Over the course of 1–2 weeks, crystals of J4 in these reproduced conditions became amorphous and dissolved, followed by the formation of crystals with a different morphology. These re-formed crystals diffracted to better than 3 Å. Initial phases obtained by Molecular Replacement using a predicted structure from SwissModel (53) revealed that the fusion loop in J4 had been cleaved, resulting in a transition of the HA from the HA0 to HA1/HA2 state. The tight packing of the HA proteins in the asymmetric unit suggested that the more compact structure facilitated better-ordered lattices. Based on this insight, TJ-2 was re-expressed and purified from insect cells. The affinity-purified protein was subsequently concentrated, then diluted 10-fold in a buffer consisting of 150 mM NaCl, 10 mM Tris (pH 7.4). The buffer-exchanged sample was supplemented with CaCl_2_ (final concentration approximately 1 mM) and then digested overnight at 4°C using trypsin at an approximately 1:1,000 mass ratio. The digested sample was concentrated, then passed through a Superdex 200 10/300 column (GE Life Sciences) equilibrated with 50 mM NaCl, 10 mM Tris (pH 7.5) to isolate intact trimeric HA from aggregates and liberated Foldon/tags. Fractions corresponding to trimeric HA1/HA2 proteins were identified and pooled based on the chromatogram and SDS-PAGE analysis, then concentrated to 10.4 mg/mL. The protein was screened as before, with crystals appearing within 3 days. The hits were reproduced manually and optimized along a precipitant gradient.

### Crystallographic data collection, structural solution, and refinement of TJ2 and J4 COBRAs

The final optimized TJ-2 crystal used for data collection was grown in a condition consisting of 0.1 M Tris pH 8.5, 21% PEG 3350. The crystal was cryoprotected in a solution consisting of 0.1 M Tris pH 8.5, 20% PEG 3350, 6% ethylene glycol, 6% DMSO, and 6% glycerol prior to flash cooling in liquid nitrogen. A data set was collected at the Advanced Photon Source, beamline 23ID-D, under cryogenic conditions. Initial data processing was performed using XDS, with final scaling and merging conducted with Aimless in the CCP4i2 interface (61, 64–68). Phases were calculated by Molecular Replacement using Phaser in the PHENIX crystallography package (57, 69) using a de-glycosylated TJ2 HA0 monomer as a search model derived from a homology model generated by SwissModel (53). Subsequent model building and refinement were performed by alternating iterations in Coot version 0.9.8.92 and PHENIX version 1.20.1-4487 (55, 57). The final model was validated with MolProbity and Privateer (58, 59). Data collection and refinement statistics are reported in Table 5. The model and structure factors have been deposited in the Protein Data Bank as entry 9B7H.

The final optimized J4 crystal used for data collection was grown in a condition consisting of 0.3 M KNO_3_, 14% PEG 3350. The crystal was immersed in a solution consisting of 0.35 M KNO_3_, 16% PEG 3350, and 20% PEG 400 for cryoprotection prior to flash cooling in liquid nitrogen. Data were collected at the Advanced Photon Source, beamline 23ID-D, under cryogenic conditions. The data were indexed, integrated, scaled, and merged using DIALS (70–72). Initial phases were calculated by Molecular Replacement in PHENIX (57, 69), with the search model consisting of a de-glycosylated J4 HA1/HA2 monomer derived from the TJ2 structure using the Sculptor program in PHENIX (57, 73). Model building and refinement were performed through alternating iterations in Coot version 0.9.8.92 and PHENIX 1.20.1-4487 (55, 57). The final model was validated with MolProbity and Privateer (58, 59). Data collection and refinement statistics are reported in Table 5. The model and structure factors have been deposited in the Protein Data Bank as entry 9B7I.

## Data Availability

All data necessary to reproduce the reported analyses are included in the main text, supplemental material, or publicly accessible databases. Models and associated data for the crystal structures have been deposited in the Protein Data Bank (www.rcsb.org) as entries 9B7H and 9B7I. Models for the single-particle cryo-EM structures have been deposited in the Protein Data Bank as entries 9B7G, 9DN2, and 9DO2, and the associated volumes in the Electron Microscopy Data Bank (www.ebi.ac.uk/emdb) as entries EMD-44305, EMD-47024, and EMD-47071.

## SUPPLEMENTAL MATERIAL

Supplemental figures jvi.00453-25-s0001.pdf Figures S1 to S8.

ASM does not own the copyrights to Supplemental Material that may be linked to, or accessed through, an article. The authors have granted ASM a non-exclusive, world-wide license to publish the Supplemental Material files. Please contact the corresponding author directly for reuse.
