# Supplementary material for: Assessing the structural boundaries of broadly reactive antibody interactions with diverse H3 influenza hemagglutinin proteins
Source: J Virol. 2025 Aug 14;99(9):e00453-25. doi: 10.1128/jvi.00453-25 (PMC12455949; doi:10.1128/jvi.00453-25)
Supplement: Supplemental figures — Figures S1 to S8. [file jvi.00453-25-s0001.pdf]

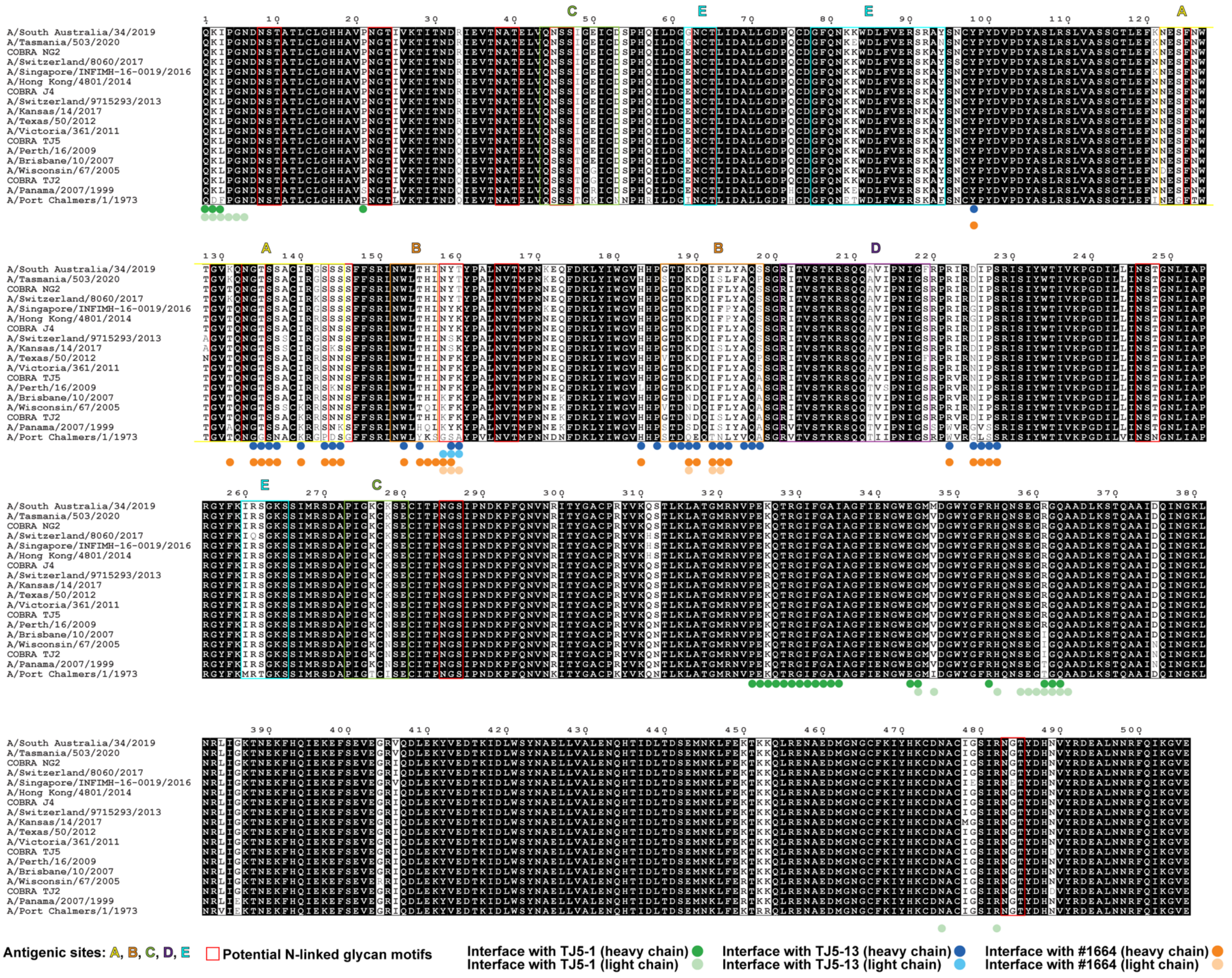

A/South Australia/34/2019
A/Tasmania/503/2020
COBRA NG2
A/Switzerland/8060/2017
A/Singapore/INFIMH-16-0019/2016
A/Hong Kong/4801/2014
COBRA J4
A/Switzerland/9715293/2013
A/Kansas/14/2017
A/Texas/50/2012
A/Victoria/361/2011
COBRA TJ5
A/Perth/16/2009
A/Brisbane/10/2007
A/Wisconsin/67/2005
COBRA TJ2
A/Panama/2007/1999
A/Port Chalmers/1/1973
Antigenic sites: A, B, C, D, E
Potential N-linked glycan motifs
Interface with TJ5-1 (heavy chain)
Interface with TJ5-1 (light chain)
Interface with TJ5-13 (heavy chain)
Interface with TJ5-13 (light chain)
Interface with #1664 (heavy chain)
Interface with #1664 (light chain)

Figure S1. Sequence alignment of the ectodomains of the H3 proteins utilized in this study. The residue positions were colored based on percentage similarity at each amin acid position by ESPript. Residues that are perfectly conserved are shown in white lettering on a black background, those that are partly conserved as bold black text on a white background, and those that are not conserved as normal black text on a white background. Potential glycosylation sites observed across these viruses are boxed in red. Antigenic sites are designated with differentially colored boxes. The residue positions that correspond to the interfaces with each antibody, based on PDBePISA, are shown.

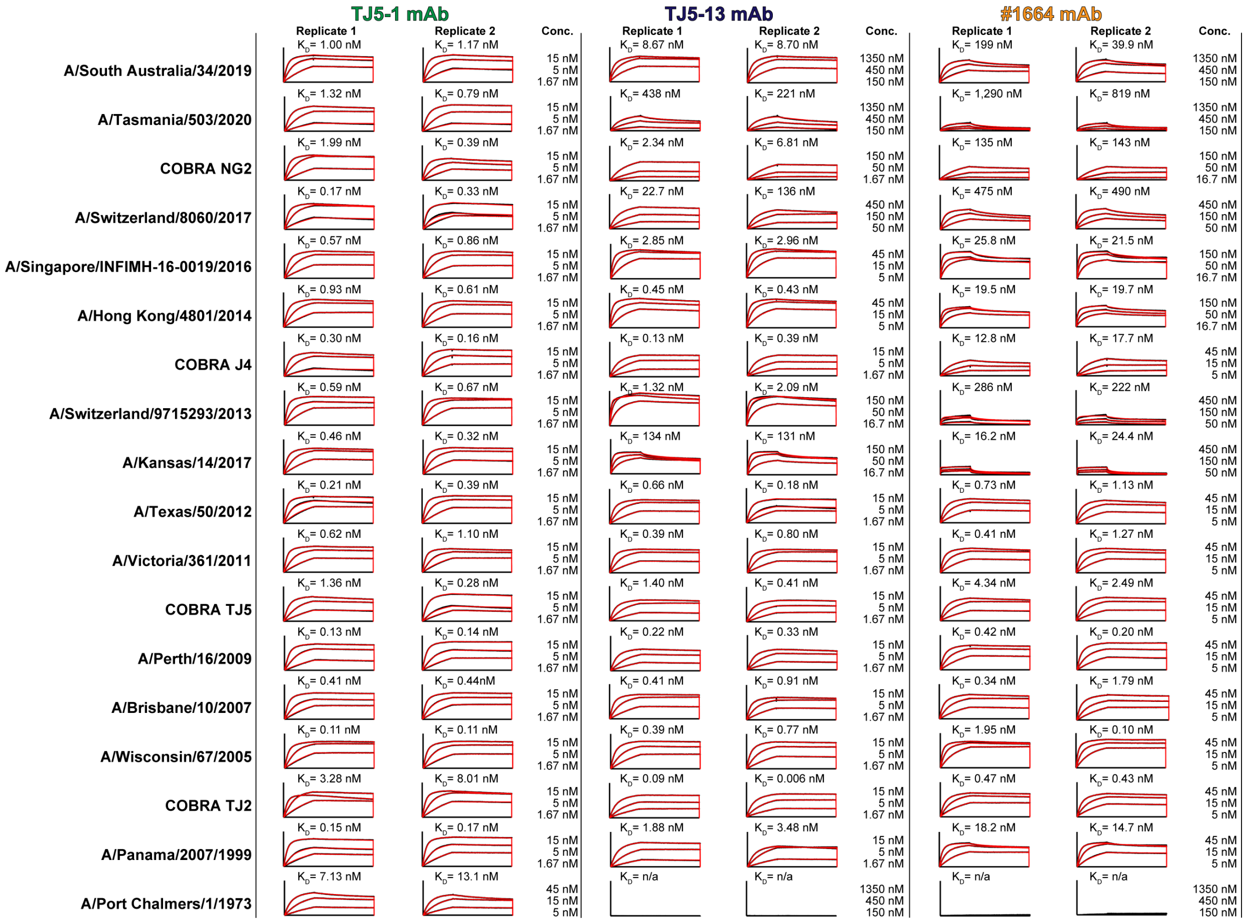

Figure S2. Binding curves for biolayer interferometry assays. The baseline adjusted and interstep corrected data during the association and dissociation stages are shown as black traces, with model fits colored red.

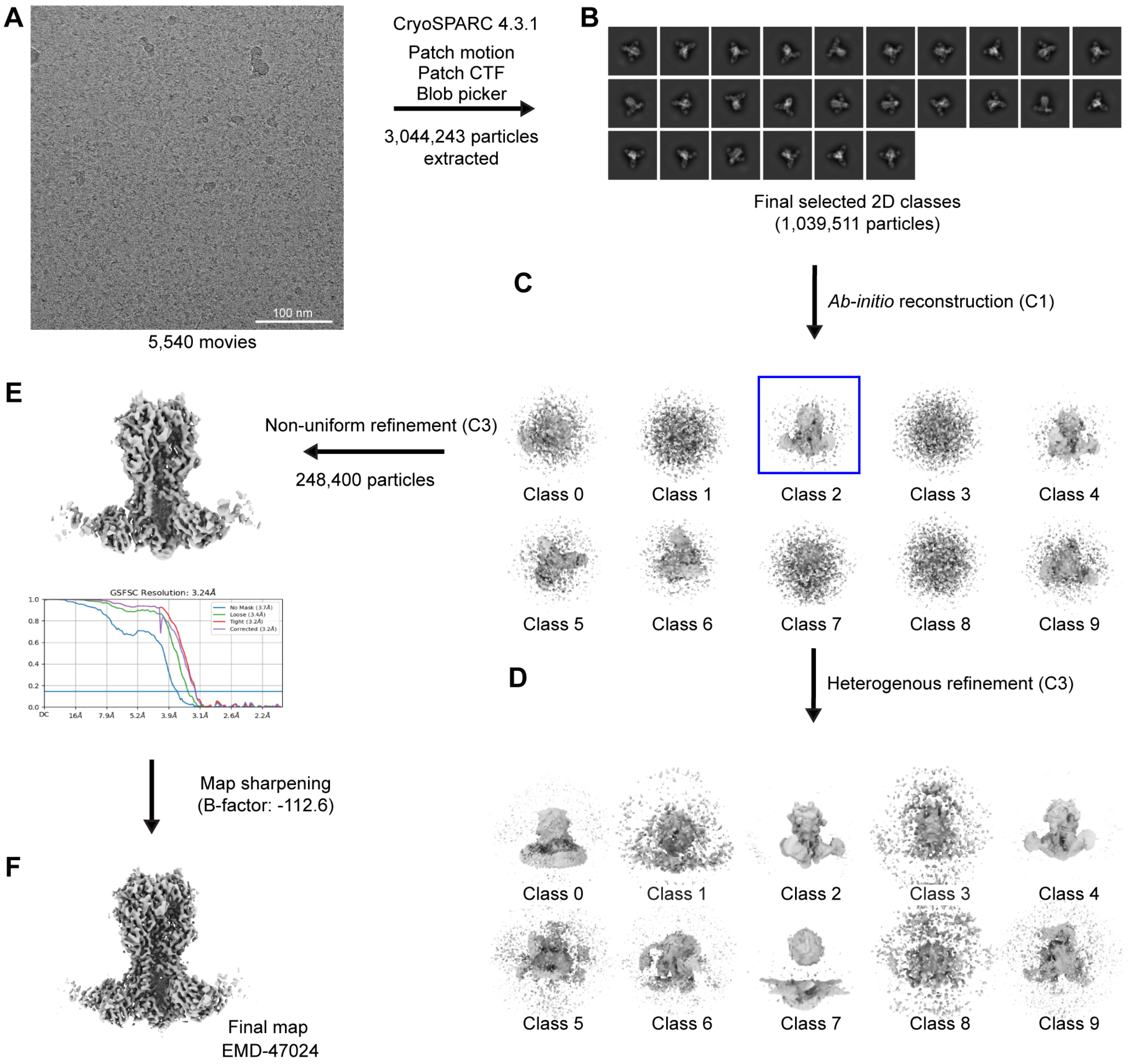

Figure S3. Cryo-EM data processing workflow for COBRA NG2:TJ5-1. (A) Sample micrograph. (B) Final set of 2D classes used for 3D reconstruction. (C) Initial 3D volumes generated by *ab-initio* reconstruction. (D) Volumes derived from heterogenous refinement. (E) Map generated from the final 3D refinement. (F) Final sharpened map used for model building.

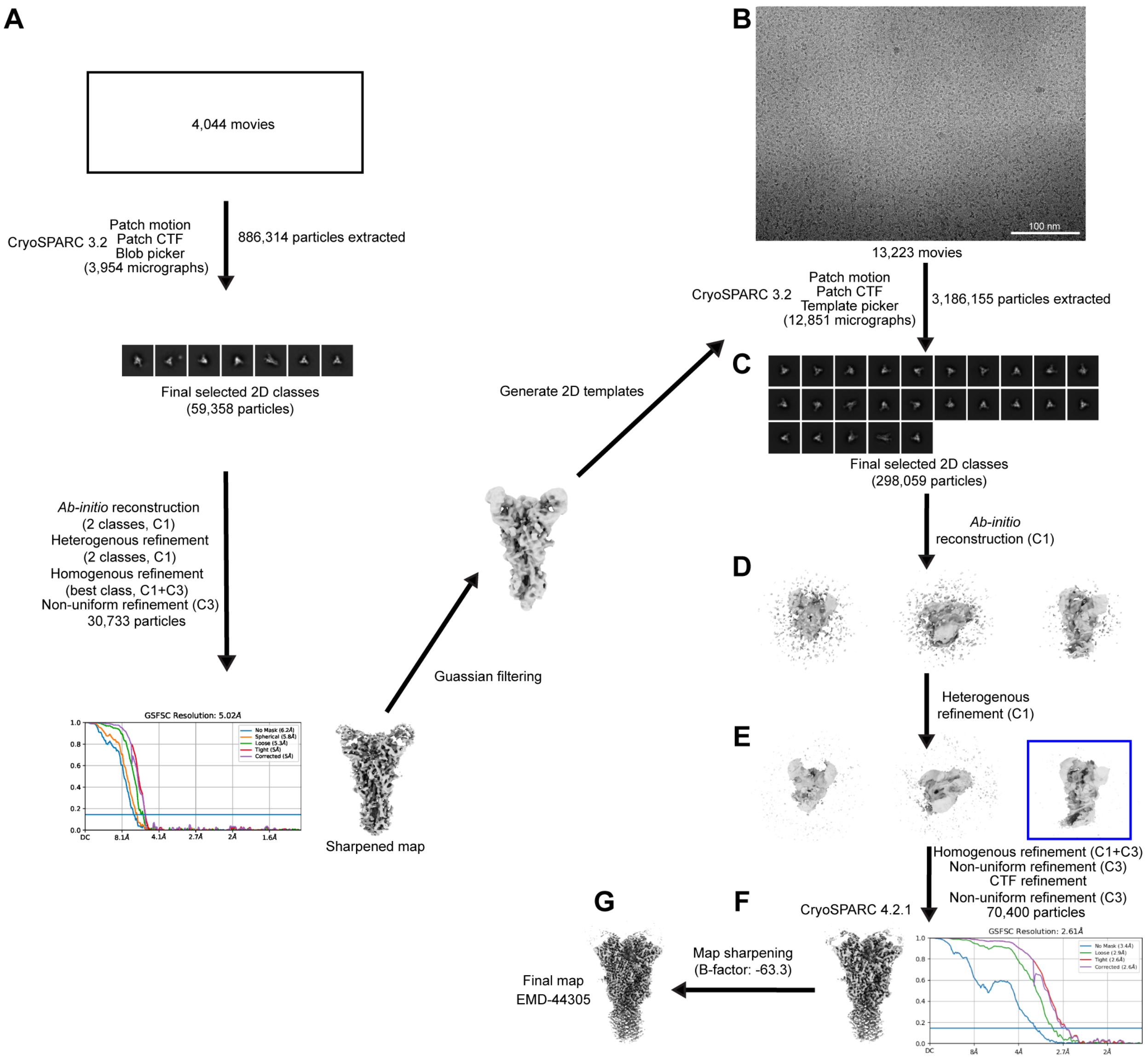

Figure S4. Cryo-EM data processing workflow for COBRA NG2:TJ5-13. (A) Abbreviated processing pipeline for the preliminary dataset used to generate a reference volume for template-based particle picking. (B) Sample micrograph of the final dataset. (C) Final set of 2D classes used for 3D reconstruction. (D) Initial 3D volumes generated by *ab-initio* reconstruction. (E) Volumes after heterogenous refinement. (F) Map and FSC curves from the final 3D refinement. (G) Final sharpened map used for model building.

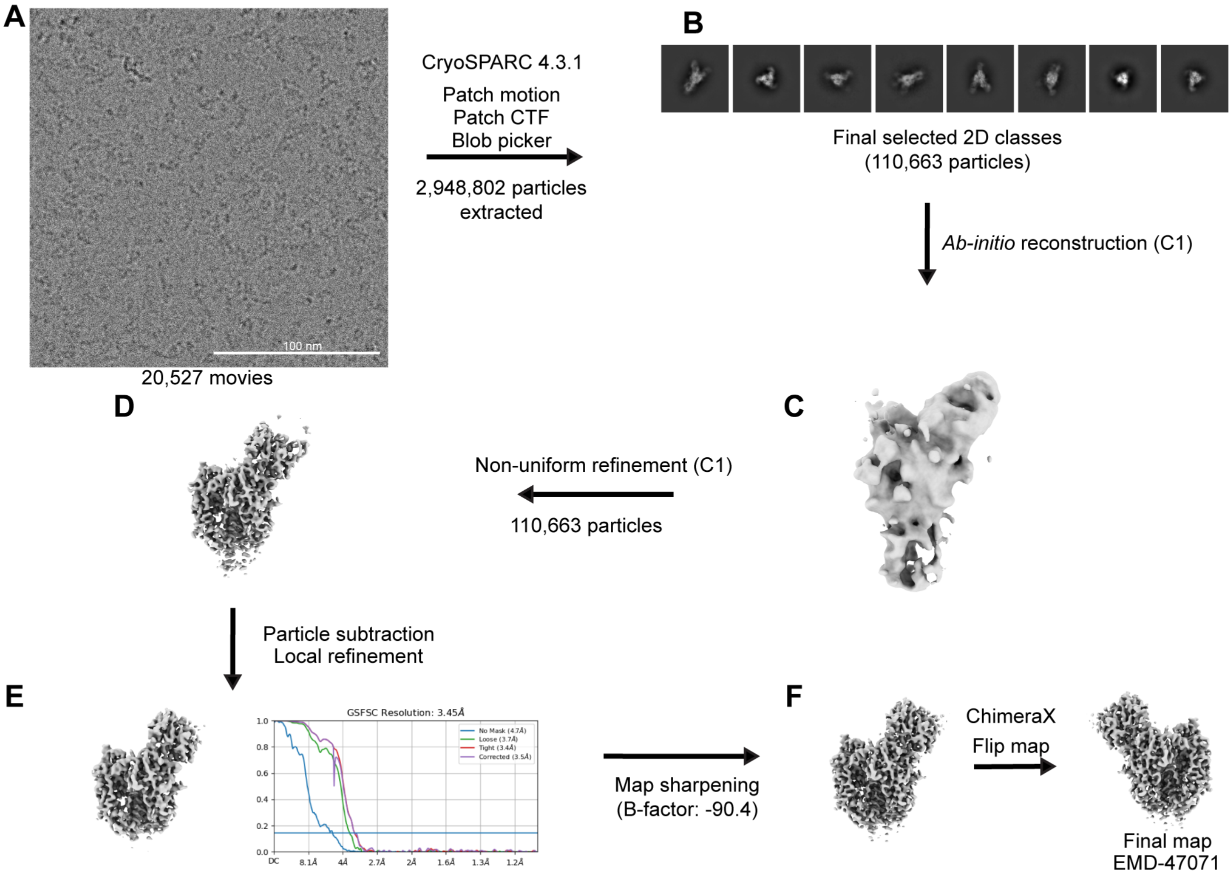

Figure S5. Cryo-EM data processing workflow for COBRA NG2:#1664. (A) Sample micrograph. (B) Final set of 2D classes used for 3D reconstruction. (C) Initial 3D volume model derived from *ab-initio* reconstruction. (D) Map after non-uniform refinement. (E) Map and FSC curves from the final Local refinement following particle subtraction. (F) The original and flipped hands of the final sharpened map used as the basis for model building.

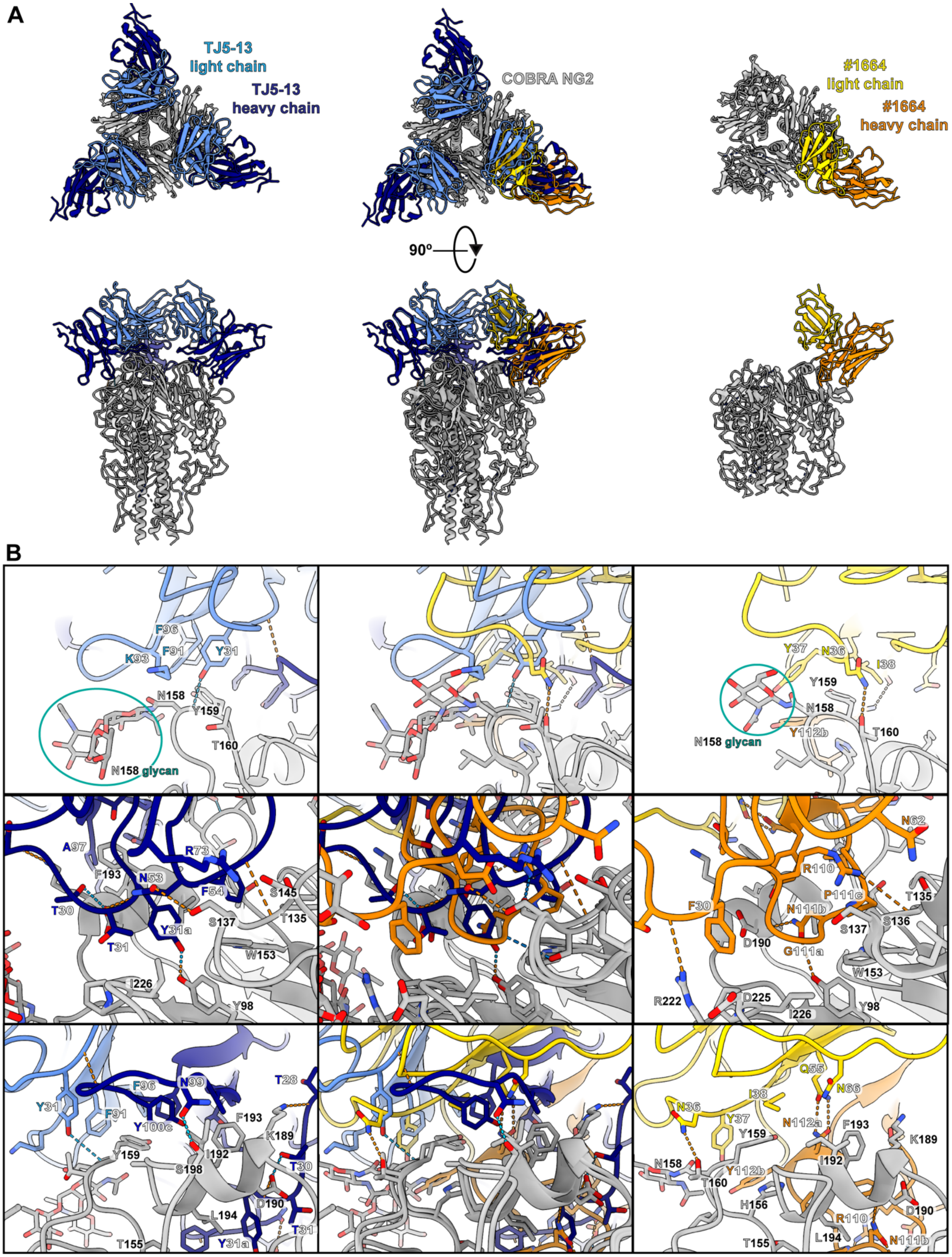
A
TJ5-13
light chain
TJ5-13
heavy chain
COBRA NG2
#1664
light chain
#1664
heavy chain
90º
B
F96
K93
F91
Y31
N158
Y159
T160
N158 glycan
Y37
N36
I38
Y159
N158
N158 glycan
Y112b
T160
A97
R73
F193
N53
F54
S145
T30
S137
T135
Y31a
T31
W153
I226
Y98
N62
R110
F30
P111c
T135
N111b
S137
S136
D190
G111a
W153
R222
D225
I226
Y98
T28
F96
N99
Y31
F91
F193
Y100c
K189
Y159
I192
S198
T30
D190
L194
T31
T155
Y31a
Q55
N66
I38
N36
Y37
N112a
F193
Y159
K189
N158
T160
Y112b
I192
H156
R110
D190
T155
L194
N111b

Figure S6. Expanded comparison of the TJ5-13 and #1664 bound COBRA NG2 structures (A) Side-by-side comparisons of the overall binding orientation of the TJ5-13 and #1664 antibodies in complex with COBRA NG2. (B) Closeup comparisons of the binding interfaces of TJ5-13 and #1664 in complex with COBRA NG2.

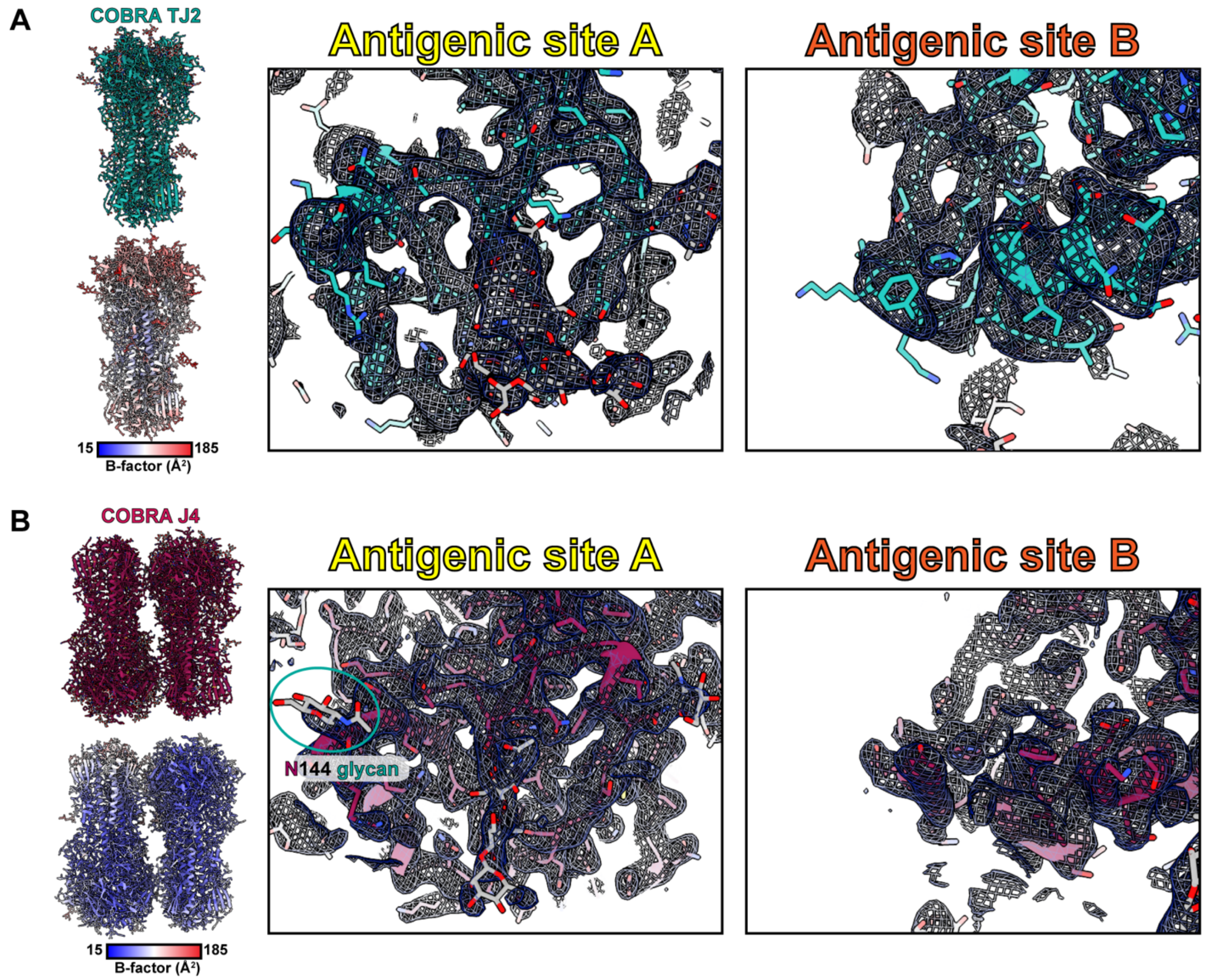

Figure S7. Additional details on the COBRA TJ2 and COBRA J4 crystal structures. (A-B) Asymmetric unit and local map regions around antigenic sites A and B for COBRA TJ2 (A) and COBRA J4 (B). The 2mFo-Fc map in CCP4 format was visualized in ChimeraX and is shown contoured at Step 1, Level 1.

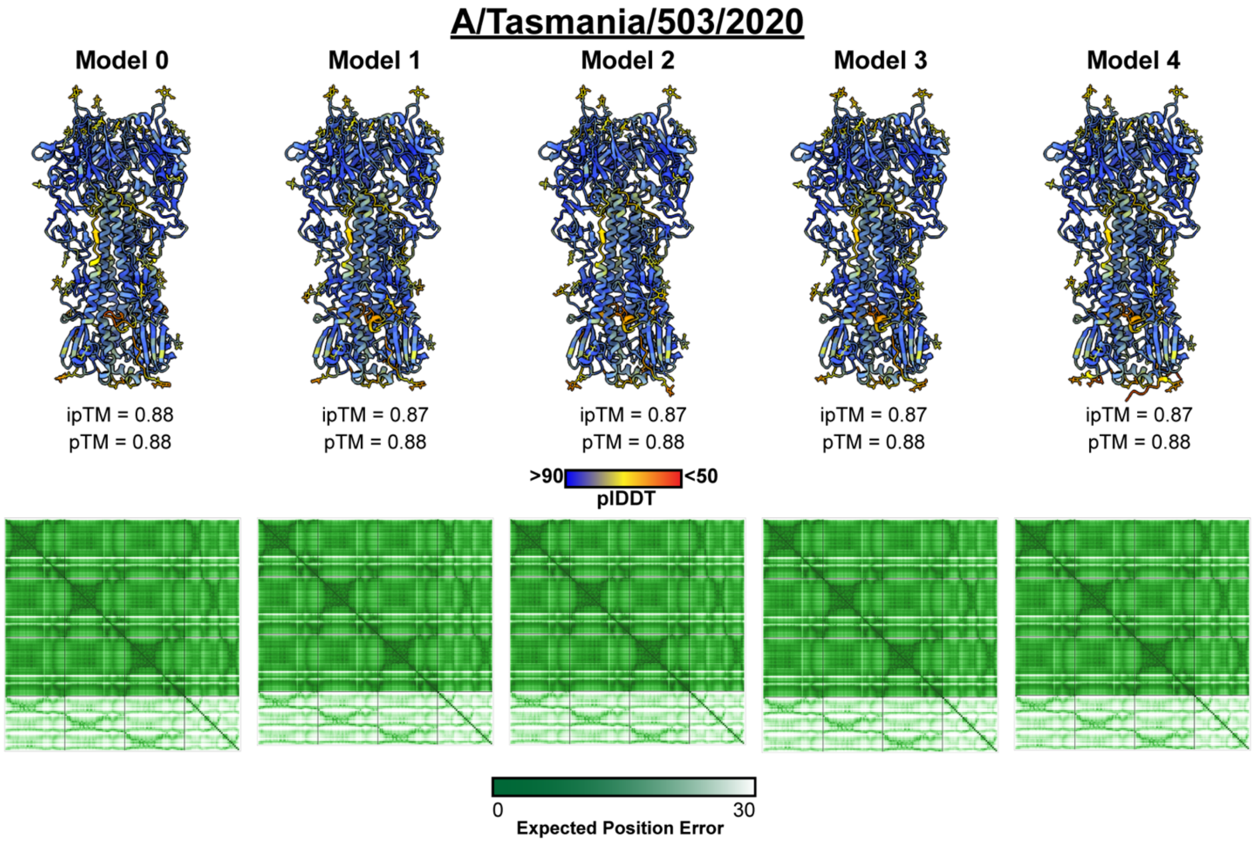

Figure S8. AlphaFold3 models and error plots for the ectodomain of A/Tasmania/503/2020. Each model and plot image was rendered in ChimeraX. Model 0 was selected for comparative analysis with other H3 proteins.
